# Curcumin in oral health: mechanisms, clinical evidence, and delivery strategies

**DOI:** 10.3389/fphar.2025.1661443

**Published:** 2026-03-02

**Authors:** Chengchen Hu, Shengguo Wang, Zhi Gao, Maofeng Qing, Lian Tan, Lu Yang, Fang Li

**Affiliations:** 1 Department of Stomatology, The Second Affiliated Hospital of Chongqing Medical University, Chongqing, China; 2 Department of General Surgery, Chongqing General Hospital, Chongqing, China

**Keywords:** anti-inflammatory, antioxidant, curcumin, drug delivery systems, oral health

## Abstract

Curcumin, a polyphenolic compound derived from the turmeric rhizome (Curcuma longa), has attracted significant interest in dentistry and oral medicine because of its multifaceted therapeutic properties. In particular, curcumin exhibits potent anti-inflammatory, antioxidant, and antimicrobial activities that are relevant to a wide spectrum of oral diseases. We conducted a narrative search of PubMed (2000–2025) using iterative keyword combinations related to curcumin and oral diseases/mechanisms, screened reference lists, and selected studies on the basis of their relevance to oral pathobiology, delivery systems, and clinical/translational outcomes. This narrative review summarized the current knowledge concerning the molecular mechanisms of curcumin and its clinical applications in oral health. We outlined how curcumin modulates key inflammatory pathways and oxidative stress responses, and how it exerts broad-spectrum antimicrobial effects against oral pathogens. We detailed the efficacy of curcumin in specific oral conditions, including periodontal diseases, dental caries, recurrent aphthous stomatitis, oral lichen planus, oral submucous fibrosis, oral candidiasis, radiation/chemotherapy-induced oral mucositis, and oral cancers. In each context, we highlighted evidence from *in vitro* studies, animal models, and clinical trials, and noted the benefits of curcumin, such as reduced inflammation, enhanced healing, microbial inhibition, and in some cases outcomes comparable to those of standard therapies. Across conditions, curcumin shows adjunctive benefit: In periodontal disease, it reduces plaque and gingival inflammation comparable to chlorhexidine and improves probing outcomes when added to scaling and root planing; in recurrent aphthous stomatitis, it reduces pain and ulcer size with steroid-like efficacy; in radiotherapy/chemotherapy-induced oral mucositis, it delays onset and decreases severity; in oral candidiasis, it decreases fungal burden and enhances photodynamic therapy; and in oral squamous cell carcinoma early clinical studies show modulation of inflammatory cytokines and the oral microbiome. Various delivery systems developed to overcome the poor bioavailability of curcumin—from mouthwashes and gels to nanocarriers and mucoadhesive formulations—are reviewed. Although many studies reported promising results with minimal toxicity or side effects, there were study limitations such as small sample sizes, variability in formulations, and the pharmacokinetic properties of curcumin. Overall, the reviewed data support the role of curcumin as a safe, formulation-dependent adjunct—not a stand-alone therapy—in oral medicine.

## 1 Introduction

**FIGURE 1:**
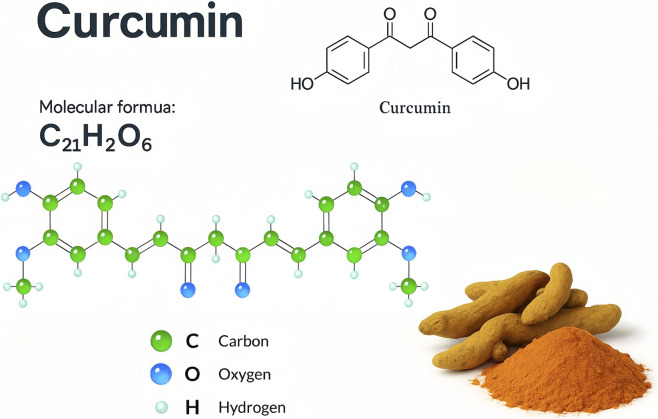
Structural formula of curcumin (diferuloylmethane), the principal bioactive polyphenol derived from *Curcuma longa*. The chemical structure of curcumin is shown in both 2D skeletal and 3D ball-and-stick models. Curcumin’s molecular formula is C_21_H_20_O_6_, with two aromatic rings connected by a seven-carbon chain containing α,β-unsaturated carbonyl groups. The phenolic hydroxyl groups and β-diketone moiety confer antioxidant and metal-chelating properties, which underlie the biological activity of curcumin. The 3D model highlights carbon (green), oxygen (blue), and hydrogen (white) atoms, and the lower right corner shows its natural plant source—turmeric rhizome powder—used in traditional medicine and functional foods.

Curcumin (diferuloylmethane) is the principal bioactive polyphenol of turmeric, a spice derived from the dried rhizome of the plant *Curcuma longa L* (family Zingiberaceae). Curcumin has a long history of use in traditional medicine and is scientifically recognized for diverse pharmacological activities. Recent studies have confirmed that curcumin has potent antioxidant, anti-inflammatory, antibacterial, and immunomodulatory properties (Stohs et al., 2020; Tabanelli et al., 2021). Chemically, curcumin consists of two phenolic rings with methoxy groups linked by a conjugated diketone bridge, conferring its ability to scavenge reactive species and bind various molecular targets (Figure 1). Clinically, the broad therapeutic potential of curcumin has been explored in conditions ranging from metabolic and inflammatory disorders (e.g., arthritis, diabetes, inflammatory bowel disease) to cancer and neurodegenerative diseases (Sohn et al., 2102; Liu et al., 2016). In the oral cavity, a wide array of diseases are driven by chronic inflammation, oxidative stress, and microbial infection, precisely the pathogenic processes modulated by curcumin. Common oral diseases such as dental caries, gingivitis, periodontitis, pulpitis, and mucosal lesions (e.g., aphthous ulcers and lichen planus) involve complex host-bacterial interactions and inflammatory cascades. Conventional treatments such as antibiotics, antiseptics, or corticosteroids can be effective but often carry side effects (e.g., antibiotic resistance, oral dysbiosis, mucosal atrophy). Thus, there is growing interest in natural adjunctive agents and botanical drugs such as curcumin that may enhance therapeutic outcomes while minimizing adverse effects (Liu et al., 2016; Sanidad et al., 2019).

Importantly, curcumin is regarded as safe and well-tolerated. It has negligible systemic toxicity and has been administered in humans at high dosages (up to 12 g/day for 3 months) without significant adverse effects (Ma et al., 2019). This safety profile, coupled with the multiple targeted mechanisms of curcumin, makes it an attractive candidate for integrative oral healthcare. However, a major challenge is its poor oral bioavailability: Curcumin is hydrophobic, unstable at physiological pH, and undergoes rapid first-pass metabolism (Tabanelli et al., 2021; Anand et al., 2007). When taken orally, 40%–85% of curcumin may pass unabsorbed through the gut. To address this, numerous delivery strategies have been investigated, including encapsulation in liposomes, nanoparticles, or controlled-release systems, as well as topical formulations tailored for the oral mucosa (Liu et al., 2016; Ipar et al., 2019).

This review provides a comprehensive overview of the role of curcumin in oral health, with emphasis on its anti-inflammatory, antioxidant, and antimicrobial actions at the molecular level, and its therapeutic applications in specific oral diseases. We also discuss the various delivery systems developed to enhance the efficacy of curcumin in the oral cavity, summarize evidence from clinical trials, and highlight future directions for research. Positioning relative to prior reviews—Prior reviews have either surveyed curcumin broadly in oral health without a formal translational framework (e.g., narrative overviews) or focused on a single indication (e.g., periodontitis or oral mucositis) or modality (e.g., curcumin-mediated photodynamic therapy). In contrast, our review is designed for oral medicine translation: We (I) tier the evidence within each disease (*in vitro* → *in vivo* → clinical) (II) emphasize delivery systems and regulatory considerations for real-world use, and (III) integrate up-to-date RCTs and systematic reviews (2022–2025). This framing complements broader or single-disease reviews (e.g., DiPalma et al., 2024; Wendorff-Tobolla et al., 2023b; Indriyani et al., 2025; Kubizna et al., 2024; Wendorff-Tobolla et al., 2023a) and aims to guide clinicians on when and how curcumin might be considered as an adjunct under evaluation rather than a routine therapy. By collating findings from high-impact studies (2000–2025), we aim to critically evaluate how curcumin could be integrated into prevention or treatment modalities for oral diseases, and what advancements are needed to realize its full potential in dentistry. To guide the reader, this review is organized as follows. Section 2 synthesizes the pharmacological mechanisms relevant to oral pathologies, focusing on (I) anti-inflammatory and immunomodulatory signaling (e.g., NF-κB, COX-2, key cytokines, and macrophage polarization) (II) antioxidant and mitochondrial pathways (e.g., Nrf2/HO-1 activation, ROS scavenging, and redox homeostasis), and (III) antimicrobial and antibiofilm actions (membrane disruption, quorum-sensing interference, and synergy with photodynamic therapy). Section 3 discusses disease-specific evidence across periodontal diseases (gingivitis/periodontitis), dental caries and endodontic infections, recurrent aphthous stomatitis, oral lichen planus, oral submucous fibrosis and leukoplakia, oral candidiasis, radiation/chemotherapy-induced oral mucositis, and oral squamous cell carcinoma. Section 4 summarizes delivery systems and formulation strategies to address bioavailability and mucosal retention (e.g., mouthrinses, gels/orabases, nanoparticles, mucoadhesive films, and photosensitizers for PDT). Section 5 synthesizes clinical trial quality, safety, and effect sizes, and Section 6 discusses translational gaps, dosing/formulation standards, and priorities for future research.

### 1.1 Search strategy and literature selection

The literature for this narrative review was identified through a comprehensive search of the electronic databases PubMed/MEDLINE, Scopus, Web of Science, and the Cochrane Library for relevant articles published between January 2000 and June 2025. The search strategy employed a combination of keywords and Medical Subject Headings (MeSH) terms related to the core concepts (“curcumin” OR “diferuloylmethane” OR “turmeric”) AND (“oral health” OR “periodontitis” OR “oral mucositis” OR “oral cancer” OR “oral lichen planus” OR “recurrent aphthous stomatitis” OR “oral submucous fibrosis” OR “dental caries” OR “oral candidiasis”) AND (“anti-inflammatory” OR “antioxidant” OR “antimicrobial” OR “mechanism” OR “drug delivery systems” OR “clinical trial”). The initial broad search yielded more than 1,200 records.

Studies were selected for inclusion based on their relevance to the molecular mechanisms, therapeutic applications, and delivery formulations of curcumin in the context of oral diseases. The inclusion criteria were original research articles (including *in vitro*, *in vivo*, and clinical studies), high-quality systematic reviews, and meta-analyses published in English. The exclusion criteria were non-peer-reviewed publications, conference abstracts without full data, studies not focused on oral health applications, and articles for which the full text was unavailable.

The selection process involved a preliminary screening of titles and abstracts to remove duplicates and obviously irrelevant reports. The full texts of the remaining articles were then critically assessed for eligibility. The reference lists of key publications were also manually searched to identify any additional relevant studies that may have been missed in the database search. This process culminated in the inclusion of approximately 180 publications that formed the evidence base for this comprehensive review. Data from these studies were synthesized narratively to highlight the mechanisms, efficacy, and future directions of curcumin in oral medicine.

## 2 Pharmacological mechanisms and pathways of curcumin

Curcumin is often described as a pleiotropic molecule because of its ability to interact with multiple cellular targets and signaling pathways. The therapeutic effects of curcumin in oral diseases stem from three primary categories of action: Anti-inflammatory, antioxidant, and antimicrobial effects. These processes are interrelated—for instance, the reduction in oxidative stress caused by curcumin also dampens inflammation—and together contribute to the chemopreventive and healing properties of curcumin in oral tissues. Below, these mechanisms and the key molecular pathways involved are detailed.

### 2.1 Curcumin’s immunomodulatory control (NF-κB/COX-2) and pro-resolving pathways in oral inflammation

Chronic inflammation is common in periodontal disease, pulpitis, oral mucosal lesions, and even oral carcinogenesis. Curcumin’s anti-inflammatory effect is among its most potent actions and is mediated by the inhibition of pivotal signaling pathways such as nuclear factor kappa B (NF-κB). NF-κB is a transcription factor that controls the expression of numerous proinflammatory genes (the cytokines IL-1β, IL-6, and TNF-α; the enzymes COX-2 and iNOS; and adhesion molecules). Curcumin can block the activation of NF-κB by preventing the phosphorylation and degradation of its inhibitor IκB kinase (IKK) (Burge et al., 2019). In cultured cells, curcumin has been shown to inhibit cytokine-induced NF-κB activation at multiple steps, blocking IκBα degradation, nuclear translocation of NF-κB (p65/RelA), and NF-κB DNA binding, ultimately reducing downstream expression of inflammatory mediators such as interleukins and chemokines (Peng et al., 2021). By targeting an upstream signaling event (possibly at the level of NF-κB-inducing kinase), curcumin prevents the cascade that leads to NF-κB-driven gene expression (Moon, 2024). Notably, this effect has been observed across various cell types and experimental models, indicating a fundamental mechanism through which curcumin exerts anti-inflammatory activity. Core mechanisms—immunomodulation (NF-κB/COX-2 and downstream cytokines), antioxidant defense (Nrf2/HO-1 and redox homeostasis), and antimicrobial/anti-biofilm actions (including photodynamic synergy)—are active across multiple oral conditions. To minimize repetition, subsequent disease sections reference this overview and focus on clinical impact and disease-specific nuances.

Preclinical and translational data indicate that curcumin dampens NF-κB–driven cytokine cascades and actively reprograms myeloid responses in oral tissues. Chemically modified curcumin (CMC2.24) promoted M2 macrophage polarization and resolved inflammatory signaling in a diabetes-induced periodontitis model, supporting a host-modulation mechanism relevant to periodontal disease management (Deng et al., 2023). In hypercholesterolemic rats with periodontitis, systemic curcumin mitigated bone resorption and collagen degradation, which is consistent with its anti-inflammatory and osteoprotective effects (Antona et al., 2025).

In parallel, curcumin suppresses other proinflammatory pathways. It downregulates the expression or activity of enzymes such as cyclooxygenase-2 (COX-2) and lipoxygenases, thereby reducing prostaglandin and leukotriene synthesis (Nasry et al., 2018). Curcumin also inhibits the production of inflammatory cytokines (e.g., IL-6, IL-8, TNF-α) and acute-phase reactants like C-reactive protein. For example, in human gingival cells stimulated with bacterial endotoxins, curcumin significantly reduced levels of IL-6 and IL-8. This cytokine suppression contributes to curcumin’s ability to resolve inflammation in periodontal tissues and oral mucosa. Moreover, curcumin interferes with signaling cascades such as mitogen-activated protein kinases (MAPKs) and AP-1, and it can activate peroxisome proliferator-activated receptor-gamma (PPAR-γ), all of which further skew cells toward an anti-inflammatory phenotype (Davoodvandi et al., 2021).

In addition to dampening proinflammatory signals, curcumin modulates the immune response; it reduces the infiltration of neutrophils and macrophages in inflamed tissues and inhibits the release of histamine from mast cells. Curcumin can promote a shift from a pro-inflammatory M1 macrophage phenotype to a reparative M2 phenotype, aiding in tissue healing (Abdollahi et al., 2023). In T lymphocytes, compared with Th1/Th17 cytokines, curcumin tends to promote the production of Th2 and T-reg cytokines, thereby mitigating cell-mediated tissue damage. In models of oral mucosal inflammation, curcumin increased the levels of anti-inflammatory IL-10 while reducing the levels of IL-2 and interferon-gamma (Mohammadi et al., 2019). Additionally, curcumin inhibits the expression of transforming growth factor-beta (TGF-β) in patients with fibrotic oral diseases. TGF-β drives fibroblast activation and collagen deposition; by blocking TGF-β and related fibrogenic factors (e.g., connective tissue growth factor, and even pathological p53 and iNOS in precancerous fibrosis), curcumin can reduce pathological scarring in conditions such as oral submucous fibrosis (Ashrafizadeh et al., 2024). Context and dose matter: Reports of M2-like polarization with curcumin are model- and formulation-dependent. Responses vary with concentration and exposure time (biphasic effects are described in some settings), the proinflammatory stimulus (e.g., LPS vs. cytokine cocktail), cell source (primary vs. immortalized), and the delivery system (native curcumin vs. analogs/nanocarriers). Several studies have shown partial or mixed phenotypes rather than a uniform M2 shift. We therefore qualify macrophage-phenotype claims and emphasize standardized dosing and phenotyping in primary human cells and *in vivo* models. Assay-dependent variability: Nrf2 activation, ROS scavenging, antibiofilm effects, and aPDT activity are concentration-, time-, and context-dependent. At higher concentrations, pro-oxidant or cytostatic effects can occur; aPDT efficacy depends on the photosensitizer concentration and light parameters; and antibiofilm activity varies with species consortia and biofilm maturity.

**FIGURE 2:**
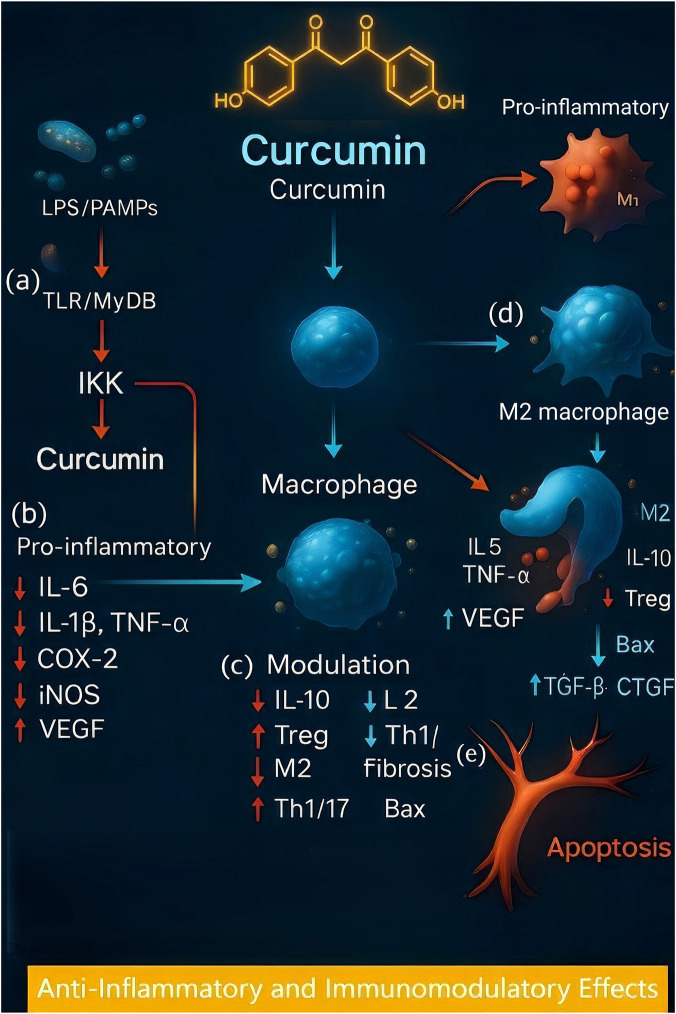
Anti-inflammatory and immunomodulatory mechanisms of curcumin in chronic oral inflammatory conditions. This schematic diagram shows how curcumin interferes with key inflammatory pathways and immune processes to alleviate chronic oral inflammation. **(a)** Upon a microbial challenge, molecules like LPS (lipopolysaccharide) or other PAMPs (pathogen-associated molecular patterns) bind to TLRs (Toll-like receptors) on immune cells and trigger a MyD88-dependent signaling cascade. This leads to activation of IKK (IκB kinase), which would normally phosphorylate the inhibitor IκBα and mark it for degradation–freeing NF-κB (nuclear factor kappa B) to translocate into the nucleus. **(b)** Curcumin is depicted blocking IKK, thereby preventing IκBα degradation. As a result, NF-κB (p65 subunit) remains sequestered in the cytoplasm and cannot induce pro-inflammatory genes. The figure highlights that curcumin’s action reduces the production of multiple inflammatory mediators, including cytokines interleukin-6 (IL-6), IL-1β (interleukin-1 beta), TNF-α (tumor necrosis factor alpha), as well as enzymes like COX-2 (cyclooxygenase-2) and iNOS (inducible nitric oxide synthase), and the angiogenic factor VEGF (vascular endothelial growth factor). **(c)** In parallel, curcumin inhibits other pro-inflammatory routes such as the MAPK (mitogen-activated protein kinase) pathways and activates the anti-inflammatory transcription factor PPAR-γ (peroxisome proliferator-activated receptor gamma), further dampening inflammatory signals. **(d)** At the cellular level, the illustration shows curcumin shifting immune responses: it drives macrophages from a pro-inflammatory M1 phenotype to an anti-inflammatory M2 phenotype, reduces Th1/Th17 (T helper type 1 and 17) responses, and enhances Treg (regulatory T cell) activity along with the release of the anti-inflammatory cytokine IL-10 (interleukin-10). Curcumin also counteracts fibrosis by inhibiting the TGF-β (transforming growth factor beta) and CTGF (connective tissue growth factor) signaling axis, thereby preventing excessive scar tissue formation. Additionally, curcumin promotes the elimination of hyperactive immune or epithelial cells by upregulating Bax (a pro-apoptotic protein) and downregulating Bcl-2 (an anti-apoptotic protein), which induces apoptosis in those cells. **(e)** Together, these mechanisms (indicated by the red inhibitory bars and green activation arrows in the figure) illustrate how curcumin resolves chronic inflammation and helps halt fibrotic changes and carcinogenic progression in oral tissues.

At higher concentrations, curcumin also induces apoptosis in activated immune and inflammatory cells, which can help terminate chronic inflammation. It upregulates pro-apoptotic factors (e.g., Bax) and downregulates survival signals (e.g., Bcl-2 and survivin) in these cells, leading to programmed cell death of hyperactivated lymphocytes or aberrant oral keratinocytes. This is relevant not only for inflammatory control but also for anticancer effects. Curcumin’s ability to inhibit angiogenesis *via* factors such as vascular endothelial growth factor (VEGF) is another aspect of its anti-inflammatory and anticancer effects. By blocking new blood vessel formation, curcumin can deprive chronic inflammatory tissue or tumors of nutrients, thereby aiding in resolution (Peng et al., 2021). The major immunomodulatory and anti-inflammatory mechanisms of curcumin are summarized in Figure 2. Evidence basis: Conceptual synthesis (hypothesis-generating); summarizes mechanisms proposed across sources without pooled quantitative validation. This schematic highlights how curcumin disrupts key inflammatory cascades such as the IKK/NF-κB pathway, shifts immune cell phenotypes toward anti-inflammatory states, suppresses the expression of pro-fibrotic factors such as TGF-β and CTGF, and promotes the apoptosis of inflammatory cells. These actions underlie the therapeutic effects of curcumin across a range of oral diseases characterized by chronic inflammation and immune dysregulation.

### 2.2 Curcumin’s antioxidant properties and ROS modulation in oral tissue homeostasis

Oxidativestress–an imbalance between reactive oxygen species (ROS) production and antioxidant defenses–is implicated in oral pathologies ranging from periodontal tissue destruction to mucosal inflammatory lesions and oral carcinogenesis. Curcumin has well-documented antioxidant capabilities, both as a direct free radical scavenger and as an inducer of the body’s antioxidant defense systems (Shahcheraghi et al., 2021). The chemical structure of curcumin (with phenolic OH groups and β-diketone moiety) allows it to directly neutralize ROS such as hydroxyl radicals, superoxide anions, and peroxyl radicals. By donating electrons or hydrogen atoms, curcumin can terminate free radical chain reactions, thus protecting cellular lipids, proteins, and DNA from oxidative damage (Cui et al., 2025).

Across oral and periodontal models, curcumin activates cytoprotective antioxidant pathways (e.g., Nrf2/HO-1) and restores redox balance, limiting downstream tissue injury. In ligature-induced periodontitis, curcumin reversed ferroptosis-linked lipid peroxidation by upregulating SLC7A11/GPX4 and reducing ACSL4/TfR1, thereby preserving gingival antioxidant capacity and attenuating alveolar bone damage (Wang et al., 2023).

In addition to this direct scavenging, curcumin upregulates endogenous antioxidant enzymes through the activation of the Nrf2 pathway. Nuclear factor erythroid 2-related factor 2 (Nrf2) is a transcription factor that, upon activation, translocates to the nucleus and binds antioxidant response elements (AREs) in DNA to drive expression of antioxidant genes. Curcumin has been shown to activate Nrf2, likely by disrupting its inhibitor Keap1 or *via* phosphorylation events, leading to increased transcription of heme oxygenase-1 (HO-1), glutathione S-transferases, NAD(P)H: quinone oxidoreductase 1 (NQO1), superoxide dismutase (SOD), catalase (CAT), and glutathione peroxidase (GPx) (Dai et al., 2023; Lee et al., 2021). By promoting the release of antioxidant enzymes such as SOD, CAT, GPx, and elevating intracellular glutathione levels, curcumin enhances the cellular capacity to detoxify ROS and reactive nitrogen species. For instance, in models of oral mucositis, curcumin’s activation of Nrf2 was associated with boosted SOD and GPx activity in mucosal cells, which correlated with reduced oxidative injury and faster healing (Dipalma et al., 2024).

Curcumin also helps maintain levels of key non-enzymatic antioxidants in tissues. In patients with oral premalignant lesions (leukoplakia, submucous fibrosis), salivary and serum assays revealed that curcumin therapy significantly increased the levels of vitamins C and E, two vital antioxidant vitamins, while reducing markers of lipid peroxidation like malondialdehyde (Jiang et al., 2020). This suggests that curcumin not only provides its antioxidant effect but may also regenerate or spare other antioxidants in the body. By preventing the depletion of vitamin C and E and inhibiting the chain reactions that lead to lipid peroxidation in cell membranes, curcumin protects oral tissues from oxidative stress–induced damage. This is particularly beneficial in inflammatory environments (e.g., chronic periodontitis or lichen planus lesions) where excessive ROS from neutrophils or damaged mitochondria can perpetuate tissue injury.

An important consequence of curcumin’s antioxidant action is the interruption of oxidative stress-inflammation cycles. Oxidative stress can activate NF-κB and other inflammatory pathways, while inflammation further generates ROS–a vicious cycle in diseases like periodontal disease. Curcumin, by concurrently reducing ROS and inflammatory signals, breaks this cycle. For example, curcumin’s activation of Nrf2 leads to higher HO-1, which not only scavenges ROS but also produces anti-inflammatory molecules like bilirubin. There is cross-talk between Nrf2 and NF-κB; curcumin’s dual effect tilts the balance toward an anti-inflammatory, antioxidant state (Cui et al., 2025). This has implications for cancer prevention as well: chronic oxidative stress and inflammation create a milieu for DNA damage and mutations. By reducing both, curcumin can help prevent the initiation and progression of oral cancer. Studies in oral cancer models showed that inhibiting Nrf2 worsens oxidative damage and inflammation, whereas curcumin’s activation of Nrf2, alongside NF-κB inhibition, lowers mutagenesis and tumor growth (Huang and Pan, 2020).

**FIGURE 3:**
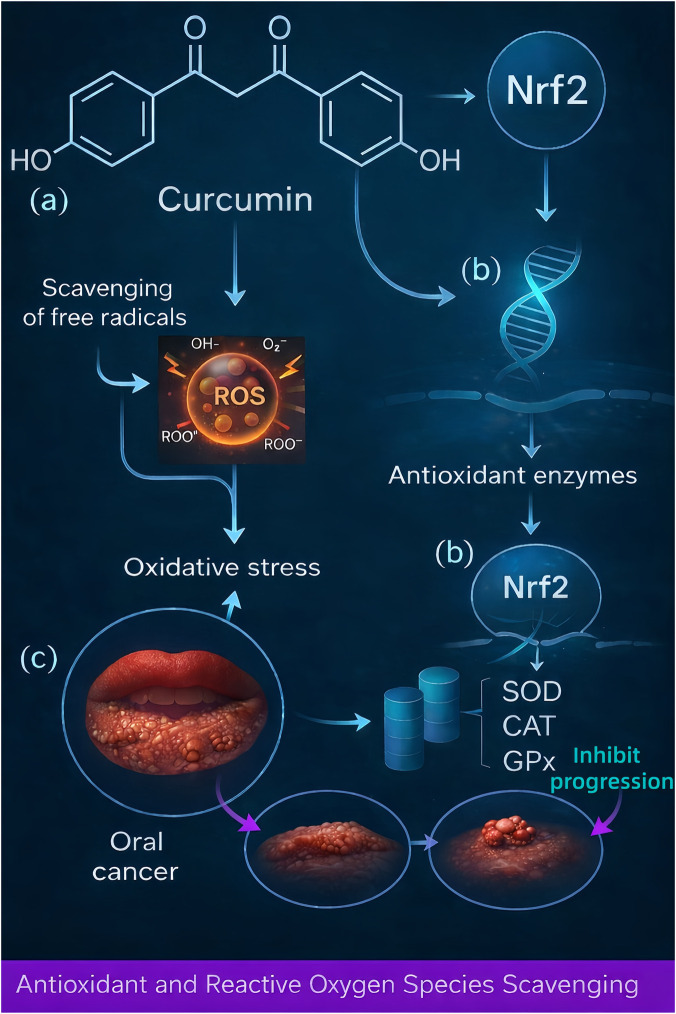
Curcumin attenuates oxidative stress in oral tissues *via* direct scavenging of reactive oxygen species and activation of Nrf2-mediated antioxidant defenses. This schematic illustration depicts two complementary antioxidant actions of curcumin that protect oral tissues. **(a)** Direct free-radical scavenging: Curcumin’s unique chemical structure (with phenolic hydroxyl groups and β-diketone moiety) allows it to neutralize reactive oxygen species (ROS) such as hydroxyl radicals and superoxide anions. By directly quenching these harmful free radicals (shown as thunderbolt symbols in the figure), curcumin reduces oxidative stress and prevents damage to lipids, proteins, and DNA in oral cells. **(b)** Nrf2 pathway activation: Curcumin simultaneously activates the Nrf2 pathway, depicted by an arrow from curcumin to the cell’s nucleus. Nrf2 (nuclear factor erythroid 2–related factor 2) is a transcription factor that, when activated, moves into the nucleus and binds to antioxidant response elements in DNA. The figure illustrates that curcumin-induced Nrf2 activation leads to elevated transcription of antioxidant enzyme genes. As a result, cells increase their levels of key antioxidant enzymes—SOD (superoxide dismutase), CAT (catalase), and GPx (glutathione peroxidase) are specifically indicated, which bolster the cell’s defense against ROS. **(c)** By both directly scavenging ROS and boosting the cell’s own antioxidant enzymes, curcumin markedly reduces oxidative injury in the oral epithelium. The downstream benefit of these actions is also shown: curcumin’s protection inhibits the progression of ROS-induced oral epithelial damage and carcinogenesis. In the figure, the development of oral cancer is represented by a sequence of changes in the mucosal layer (from epithelial thickening or hyperplasia to the formation of nodular tumors). Curcumin is shown to interrupt this sequence, thereby helping to prevent the transformation of normal oral mucosa into a cancerous lesion under conditions of chronic oxidative stress.

Overall, curcumin serves as an antioxidant guardian in the oral cavity–neutralizing existing free radicals, upregulating protective antioxidant proteins, and thereby preserving cellular integrity. These properties underlie its efficacy in mitigating tissue destruction in periodontal disease, reducing oral mucosal erythema and ulceration, and protecting against the carcinogenic progression of precancerous lesions by preventing oxidative DNA damage. The mechanistic pathway of curcumin’s antioxidant effects is visually summarized in Figure 3. This diagram highlights how curcumin not only neutralizes ROS through direct scavenging but also enhances endogenous antioxidant defenses *via* Nrf2 activation. These dual actions contribute to the suppression of oxidative injury and support oral tissue resilience under inflammatory or carcinogenic stress.

### 2.3 Broad-spectrum antimicrobial and anti-biofilm actions in the oral microbiome, including PDT synergy

Curcumin exhibits a broad spectrum of antimicrobial activity that is highly relevant to oral health, given that oral diseases are often polymicrobial. It has demonstrated inhibitory effects against Gram-positive bacteria, Gram-negative bacteria, and fungi commonly found in the oral environment (Kubizna et al., 2024; Mohammadi et al., 2019). Unlike conventional antibiotics, curcumin’s antimicrobial action is multi-modal and less prone to inducing resistance, making it an appealing adjunct or alternative, especially in an era of rising antibiotic resistance. Mechanistically, curcumin disrupts oral biofilms and enhances light-activated killing (aPDT) of key pathogens implicated in caries and periodontal disease. Randomized clinical data in orthodontic patients show that nanomicelle curcumin–mediated aPDT reduced *Streptococcus mutans* virulence and white-spot lesion dynamics *versus* controls (Nader et al., 2022). *In vitro* and translational work also documents curcumin-aPDT activity against *Porphyromonas gingivalis* biofilms and *Candida* spp., with emerging combinations (e.g., curcumin + chlorhexidine) potentiating antibiofilm effects (Harris and Sulewski, 2023; da Silva et al., 2025).

#### 2.3.1 Antibacterial mechanisms

Curcumin can directly suppress the growth of cariogenic and periodontopathogenic bacteria. For instance, *S. mutans*, the primary bacterium associated with dental caries, is inhibited by curcumin in several ways. Curcumin at micromolar concentrations has been shown to inhibit *S. mutans* adherence and biofilm formation (Picco et al., 2019). Specifically, curcumin interferes with the function of *S. mutans* sortase A–an enzyme that anchors surface adhesion proteins critical for bacterial aggregation on tooth surfaces–thereby reducing the bacterium’s ability to colonize enamel. Curcumin also downregulates genes involved in extracellular polysaccharide synthesis (such as *gtf* genes encoding glucosyltransferases), sugar metabolism, and two-component regulatory systems in *S. mutans*, all of which are essential for robust biofilm production (Sanches et al., 2019). As a result, curcumin-treated *S. mutans* forms thinner, less cariogenic biofilms. In a dental caries model, a curcumin-loaded bio-nanocomposite released curcumin slowly and significantly prevented *S. mutans* biofilm formation and tooth decay (Picco et al., 2019).

Curcumin is also effective against periodontal bacteria such as *P. gingivalis* and *Aggregatibacter actinomycetemcomitans*. It can disrupt their cell envelopes and inhibit the proteases and toxins they produce. One study found curcumin’s minimum inhibitory concentration (MIC) against *P. gingivalis* was in the low μg/mL range, indicating strong antibacterial potency (Pan et al., 2020). Curcumin-treated *P. gingivalis* showed reduced fimbriae expression, which correlates with decreased ability to invade periodontal tissues.

Beyond planktonic bacteria, curcumin targets biofilms–structured bacterial communities notoriously resistant to standard antimicrobials. Curcumin can penetrate biofilms and, by generating localized oxidative stress, kill embedded bacteria. Moreover, curcumin has been reported to inhibit bacterial quorum sensing (the cell-cell communication that regulates biofilm genes) (Afrasiabi et al., 2022). For example, in *S. mutans*, curcumin affects the quorum-sensing system by which the bacteria coordinate biofilm maturation. This anti-biofilm property is beneficial in preventing dental plaque formation and in disrupting mature plaques that cause gingivitis and periodontitis.

Another intriguing mechanism is curcumin’s ability to sensitize bacteria to antibiotics. It can permeabilize bacterial membranes and inhibit efflux pumps, thereby enhancing the uptake and retention of other antimicrobial agents. A striking example is curcumin’s synergy with antibiotics against resistant organisms: curcumin greatly improved the efficacy of polymyxin and vancomycin against both Gram-negative and Gram-positive pathogens by increasing cell membrane permeability and blocking drug efflux (Afrasiabi et al., 2022). While these studies were in systemic infections, the principle applies to oral microbes–curcumin could potentiate local antiseptics or lower the required dose of antibiotics in periodontal therapy. Curcumin’s membrane-permeabilizing effect also means it disrupts bacterial cell walls/membranes on its own, causing leakage of cellular contents and bacterial death. This has been observed with *Candida* (fungal) cells as well–curcumin interacts with membrane ergosterol, akin to polyene antifungals, leading to membrane destabilization.

#### 2.3.2 Antifungal effects

Fungal infections like oral candidiasis (thrush) are common in immunocompromised individuals and denture wearers. Curcumin has demonstrated promising antifungal activity against *Candida albicans* and non-*albicans Candida* species. It inhibits the growth of even nystatin-resistant *C. albicans* strains at MIC values around 7.8–32 μg/mL (Kubizna et al., 2024), suggesting it can tackle strains that evade standard antifungals. Curcumin impairs *Candida* by multiple routes: it disrupts fungal cell walls and membranes, generating oxidative stress within the yeast cells, and it can bind to fungal heat shock proteins and enzymes, thereby hindering their stress response and virulence. Notably, curcumin also prevents *Candida* adhesion to epithelial cells and acrylic surfaces (denture material) by affecting yeast cell-surface adhesins (Harris and Sulewski, 2023). This anti-adhesive effect is critical because *Candida* biofilm formation on dentures or mucosa leads to persistent infections.

One advanced application is curcumin-mediated photodynamic therapy (PDT) for fungal infections. Curcumin is a natural photosensitizer: under blue light illumination, curcumin produces singlet oxygen and free radicals that are lethal to microbes. In a murine model of oral candidiasis, topical curcumin combined with blue light PDT significantly reduced fungal burden and improved lesions compared to untreated controls (Kubizna et al., 2024). Curcumin-PDT effectively inactivated *C. albicans in vivo* by generating ROS within the fungal cells, causing cell death without harming host tissue. This approach has potential as an alternative to systemic antifungals, especially for localized oral candidal infections.

#### 2.3.3 Antiviral and other microbial effects

Though less studied in the oral context, curcumin has shown inhibitory effects on viruses (including herpes simplex virus and others) by interfering with viral entry and replication. In oral health, this suggests possible benefits in viral ulcerative diseases (like herpes labialis or possibly oral HPV-related lesions), though clinical evidence is still emerging. Curcumin’s broad anti-infective profile even extends to certain parasites and endodontic microbes (e.g., *E. faecalis* in root canals) – for instance, curcumin added to irrigants enhances disinfection of root canal systems by breaking down biofilms of *Enterococcus faecalis*, which is often resistant to conventional irrigants.

**FIGURE 4:**
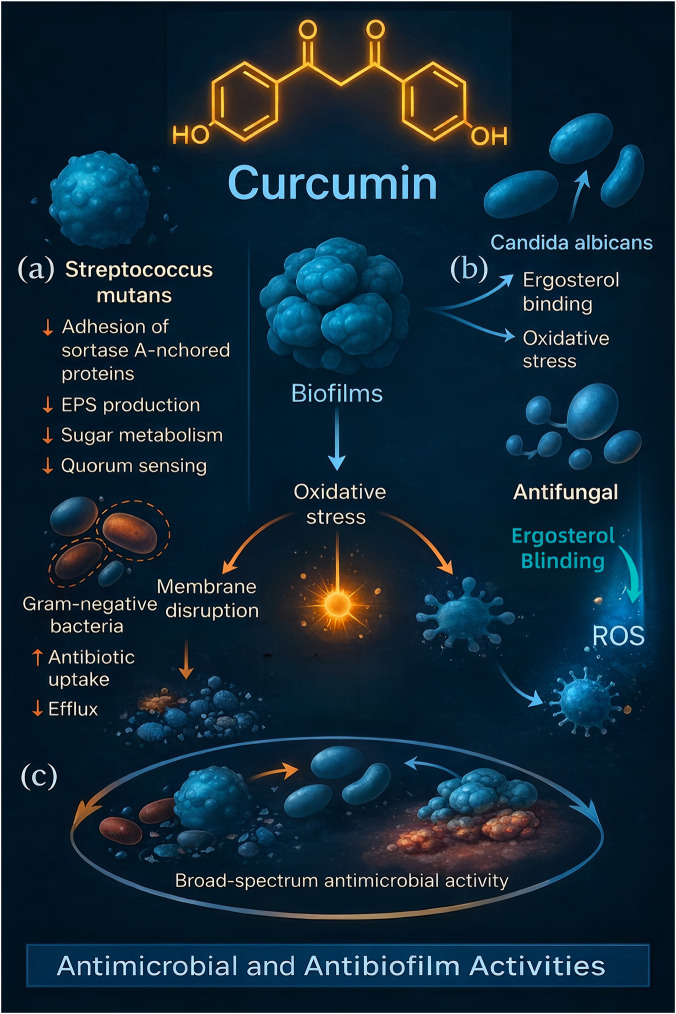
Multifaceted antimicrobial and anti-biofilm actions of curcumin relevant to oral infections. This illustration demonstrates how curcumin exerts broad-spectrum antimicrobial effects against oral pathogens, targeting both bacteria and fungi. **(a)** Anti-biofilm and antibacterial actions: For a cariogenic bacterium like *Streptococcus* mutans, curcumin interferes at multiple steps (indicated by red inhibitory lines in the figure). It inhibits the adhesion of sortase A-anchored surface proteins (preventing bacteria from attaching strongly to tooth surfaces), suppresses extracellular polysaccharide (EPS) production (limiting the sticky matrix that holds biofilms together), interferes with bacterial sugar metabolism, and downregulates quorum-sensing pathways (disrupting the cell–cell signaling needed for coordinated biofilm growth). Collectively, these actions lead to impaired dental plaque biofilm formation. Curcumin is also shown to penetrate established biofilms and generate stress within them: it produces oxidative stress inside the biofilm matrix, which helps to break apart the structured bacterial communities. Additionally, curcumin increases the susceptibility of bacteria to other antimicrobials—the figure indicates that curcumin makes bacterial membranes more permeable and inhibits drug efflux pumps, enhancing the efficacy of conventional antibiotics. It even directly damages bacterial membranes, particularly in Gram-negative bacteria (which are depicted with disrupted outer membranes), leading to leakage of cellular contents and bacterial death. **(b)** Antifungal and auxiliary effects: Against the fungal pathogen *Candida* albicans, curcumin targets the fungal cell membrane by binding to ergosterol (a key membrane sterol in fungi). This disrupts membrane integrity and induces oxidative stress inside the fungus, thereby reducing its virulence and exerting a direct fungicidal effect. Moreover, curcumin can act as a natural photosensitizer: under exposure to blue light (as illustrated by a blue light icon in the figure), curcumin molecules generate reactive oxygen species (ROS) that inactivate microbial cells. This photodynamic action is effective not only against fungal cells but also helps destroy oral viral particles if present. **(c)** Through these combined mechanisms—disrupting biofilms, damaging microbial membranes, blocking quorum-sensing signals, and working synergistically with other antimicrobials—curcumin exhibits a robust, broad-spectrum antimicrobial potential. The figure encapsulates how curcumin could be utilized as an adjunctive oral therapeutic agent to combat dental plaque bacteria, fungal infections like oral candidiasis, and other oral microbes.

The breadth of curcumin’s antimicrobial and antibiofilm effects is visually summarized in Figure 4. As shown, curcumin disrupts bacterial and fungal viability through membrane damage, oxidative stress, quorum-sensing inhibition, and biofilm disassembly. These mechanisms collectively contribute to its efficacy against oral pathogens such as *Streptococcus* mutans, *Candida* albicans, and Gram-negative bacteria, reinforcing its role as a versatile antimicrobial adjunct in oral health applications.

In summary, curcumin’s antimicrobial armamentarium includes: direct bactericidal and fungicidal activity, disruption of microbial adhesion and biofilms, inhibition of microbial virulence factors and communication, and synergy with conventional antimicrobials. These features are highly valuable in managing dental plaque, periodontal infections, endodontic infections, and oral candidiasis. By reducing microbial load and virulence while also modulating the host response (inflammation), curcumin addresses both sides of the host-microbe interplay in oral disease. This multimodal antimicrobial action makes curcumin a promising agent to incorporate into oral hygiene products (e.g., antiplaque mouthwashes, periodontal gels, denture cleansers) or to use as an adjunct in periodontal therapy and treatment of oral infections.

## 3 Translational clinical impact across oral conditions

**FIGURE 5:**
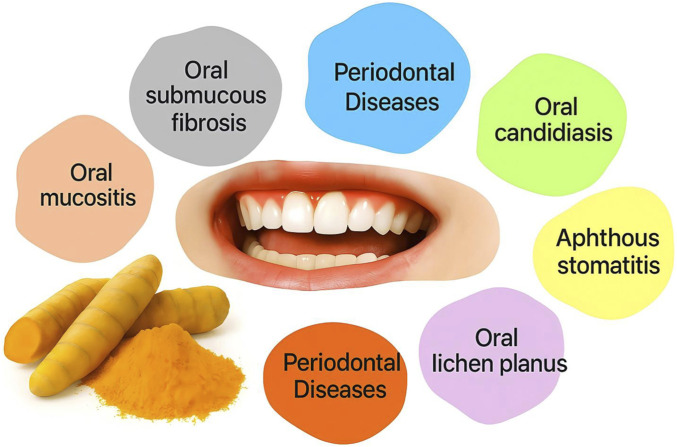
Curcumin’s importance in different clinical situations. Curcumin has been investigated as a therapeutic agent in numerous oral health conditions, including periodontal diseases (gingivitis and periodontitis), dental caries, recurrent aphthous stomatitis, oral lichen planus, oral submucous fibrosis, oral candidiasis, oral mucositis, and oral cancer. Its anti-inflammatory, antioxidant, and antimicrobial properties contribute to potential benefits in each of these conditions.

Curcumin’s combined anti-inflammatory, antioxidant, and antimicrobial effects translate into therapeutic potential across a broad range of oral diseases and conditions. Figure 5 illustrates the diverse oral conditions in which curcumin has been studied, from dental caries to oral cancer. In this section, we examine the evidence for curcumin’s efficacy in specific disease contexts, highlighting relevant *in vitro* findings, animal studies, and human clinical trials for each condition.

To provide a clear overview of how curcumin’s therapeutic potential translates across different stages of research, we first summarize the evidence hierarchy spanning *in vitro*, *in vivo*, and human clinical studies. Table 1 maps curcumin’s effects across major oral disease areas, allowing readers to visualize the consistency and translational strength of the findings before delving into each disease context in detail.

**TABLE 1:** Evidence map of curcumin’s effects across key oral disease areas (*in vitro* → *in vivo* → clinical).

Disease area	*In vitro* mechanistic (key findings and landmark refs)	*In vivo*/animal (key findings and landmark refs)	Clinical (human) — main findings, quality notes and refs
Periodontitis	Strong evidence for NF-κB and COX-2 suppression, Nrf2/antioxidant activation; direct anti-biofilm effects; curcumin as photosensitizer for aPDT in lab biofilms. Key *in-vitro* refs: Abdalla 2019 (biofilm/PDT), and mechanistic reviews. (Hormdee et al., 2025)	Multiple animal models show reduced local inflammatory markers and mitigated bone loss after topical/oral curcumin; aPDT reduces burden in ligature and infected models. Representative refs (Hormdee et al., 2025).	Multiple small RCTs and several recent systematic reviews/meta-analyses show modest but statistically significant adjunctive gains (PPD/CAL) for local curcumin formulations vs. SRP alone; heterogeneity in formulations, short follow-up, and RoB concerns temper interpretation. Key syntheses: meta-analysis (2022) and systematic reviews (Wendorff-Tobolla et al., 2023a).
Oral mucositis (RT/CT)	*In vitro*: curcumin protects mucosal cell lines from oxidative stress; modulates NF-κB/Nrf2 and cytokine release in epithelial cultures. Key refs in mechanistic reviews (Inchingolo et al., 2024).	Preclinical chemo/RT mucositis models: reduced ulceration and inflammatory markers with topical or systemic curcumin formulations ( Ramezani et al., 2023 ).	Multiple RCTs and two recent systematic reviews/meta-analyses report reduced incidence/severity and pain (WHO/OMAS grades) with curcumin mouthwashes/lozenges/gels compared with placebo or standard care; formulation heterogeneity and small sample sizes remain limiting (Dipalma et al., 2024).
Oral candidiasis/aPDT	*In vitro*: curcumin shows antifungal and anti-biofilm activity vs. *Candida* spp.; effective as a blue-light photosensitizer in biofilm eradication (Balakrishnan and Lee, 2024).	Animal and *ex vivo* denture biofilm models confirm reduction in fungal burden with curcumin-PDT protocols (Balakrishnan and Lee, 2024).	Clinical data are limited: small pilot clinical studies/case series suggest aPDT with curcumin may reduce fungal burden in denture stomatitis or refractory candidiasis, but head-to-head RCTs vs. nystatin/azole are scarce; thus, clinical application remains adjunctive/experimental (Dipalma et al., 2024)
Oral premalignant lesions (OLP/OSF/Leukoplakia)	*In vitro*: antiproliferative, anti-inflammatory actions; modulation of pro-inflammatory cytokines and oxidative stress markers in oral keratinocyte models (Inchingolo et al., 2024).	Selected animal/preclinical models show a reduction in dysplasia markers or mucosal inflammation with topical/systemic curcumin (Indriyani and Nur’aeny, 2025)	Clinical evidence is mainly small, pilot, or uncontrolled trials showing symptomatic relief and occasional histologic or biomarker changes. No trials demonstrate prevention of malignant transformation; long-term biopsy-correlated follow-up is lacking. Quality and size limitations noted (Indriyani and Nur’aeny, 2025).
Oral squamous cell carcinoma (OSCC)	*In vitro*: strong anti-proliferative, pro-apoptotic, anti-invasive effects; impacts on NF-κB, STAT3, apoptosis pathways in OSCC cell lines (Inchingolo et al., 2024).	Xenograft models show tumor-growth inhibition and cytokine modulation with systemic or local curcumin formulations (Inchingolo et al., 2024).	Early-phase human studies and window trials (e.g., APG-157 — botanical curcuminoid lozenge) show salivary cytokine and microbiome modulation and measurable exposure; no demonstrated survival or local-control benefit to date. APG-157 has received recent regulatory attention (Fast Track). Clinical use limited to trials (Inchingolo et al., 2024).

Abbreviations: CHX, chlorhexidine; PDT, photodynamic therapy; aPDT, antimicrobial photodynamic therapy; PPD, probing pocket depth; CAL, clinical attachment level; WHO/OMAS, mucositis grading scales. The table highlights direction and evidence level; readers should cross-check the cited systematic reviews and representative.

### 3.1 Periodontal diseases—adjunct to SRP: plaque/gingival reductions and probing-depth/attachment gains

Periodontal diseases, including gingivitis and periodontitis, are chronic inflammatory infections of the gingiva and supporting periodontal structures caused by dental plaque biofilms. They are among the most prevalent oral diseases globally. Given curcumin’s antiplaque and anti-inflammatory actions, it has been extensively studied as an adjunct in periodontal therapy. Clinical adjunctive use is supported by randomized trials and meta-analyses. A 2025 randomized clinical study in smokers with chronic periodontitis found that adding 2% subgingival curcumin gel to SRP produced greater PPD reduction and CAL gain at 8 weeks compared with SRP alone (Gupta et al., 2025). Two recent meta-analyses concluded that local curcumin as an adjunct to SRP yields statistically significant, albeit modest, improvements in CAL and PPD compared with SRP alone and comparable outcomes to chlorhexidine adjuncts (Wendorff-Tobolla et al., 2023b).

Most periodontal RCTs are single-center with modest sample sizes and short follow-up (often ≤8–12 weeks), and formulations/doses vary across studies. Current evidence supports curcumin primarily as a therapeutic adjunct to scaling and root planing (SRP) rather than as stand-alone prevention. Data for primary prevention of periodontitis (beyond routine oral hygiene) are limited, and long-term maintenance benefits remain unproven.

#### 3.1.1 Antiplaque and antigingivitis effects

Several clinical trials have shown that curcumin-based mouthwashes can reduce dental plaque and gingival inflammation comparably to chlorhexidine (the gold-standard antiseptic mouthrinse). In a double-blind randomized trial, a 0.1% curcumin mouthwash used twice daily significantly reduced plaque index (PI), gingival index (GI), and bleeding index over 4 weeks, with efficacy statistically equivalent to 0.2% chlorhexidine and superior to placebo (Terby et al., 2021). Curcumin and chlorhexidine groups both achieved ∼60–65% reduction in plaque and gingivitis scores, whereas the placebo saw minimal improvement. Notably, curcumin mouthwash lacked the side effects of chlorhexidine (e.g., tooth staining, taste disturbance), offering a safer prophylactic rinse.

Meta-analyses have echoed these findings: curcumin mouthwashes lead to significant reductions in PI and GI, with no statistically significant difference from chlorhexidine (Oliveira et al., 2021). For instance, one review (Zhang et al., 2022) reported that curcumin mouthrinse reduced GI by about 0.4–0.5 on a 0–3 scale, nearly identical to chlorhexidine, both clearly outperforming placebo. Curcumin’s efficacy stems from its antibacterial effects against plaque biofilms and its anti-inflammatory action that reduces gingival redness and bleeding. Patients often prefer curcumin rinses due to a milder flavor and lack of burning. Most mouthwash trials to date are single-center with modest sample sizes and short follow-up (commonly 2–4 weeks), and risk-of-bias elements vary (e.g., allocation concealment and outcome-assessor blinding not always clearly reported). Formulations and comparators are heterogeneous (curcumin 0.05%–1.0% vs. chlorhexidine 0.12%–0.2%, differing rinse volumes/frequencies), and adverse-event reporting is inconsistent. These factors may influence effect estimates and limit generalizability.

#### 3.1.2 Adjunctive use in periodontitis

In chronic periodontitis, the standard care is mechanical debridement *via* scaling and root planing (SRP). Adjunctive curcumin, delivered as a gel or rinse, improves outcomes. For example, a randomized controlled trial (RCT) found that applying curcumin gel into periodontal pockets after SRP significantly reduced probing depth and gingival inflammation, and lowered IL-1β in crevicular fluid compared to SRP alone (Mohammad, 2020). Another RCT found similar outcomes with reduced inflammatory markers and faster symptom resolution.

A systematic review and meta-analysis by Zhang et al. (2022), covering 18 RCTs and 846 patients, found that curcumin adjunct therapy consistently improved periodontal indices *versus* SRP alone. GI reductions ranged from −0.2 to −0.5, sulcus bleeding index decreased at all time points, and probing pocket depth (PPD) reductions were significantly enhanced. Some studies showed improvements in clinical attachment level (CAL) and decreased systemic inflammatory markers such as salivary procalcitonin (Abdel-Fatah et al., 2023). Across the included RCTs, heterogeneity in delivery systems (gel, rinse, chips), dosing schedules, and follow-up windows (typically 4–12 weeks) is substantial; many trials are small and single-center with unclear or high risk of bias in at least one domain. Meta-analytic gains are therefore interpreted as modest, formulation-dependent adjunctive effects rather than definitive superiority, underscoring the need for standardized curcumin preparations, harmonized dosing, and longer blinded trials.

#### 3.1.3 Microbiological and delivery insights

Curcumin’s antimicrobial actions also aid therapy. Subgingival application suppresses pathogens like *Porphyromonas gingivalis* and *Tannerella forsythia*. A clinical trial using 1% curcumin gel as a subgingival irrigant or chip significantly reduced red-complex bacteria, yielding microbial results comparable to chlorhexidine chips (Inchingolo et al., 2024). Sustained-release curcumin chips inserted into deep pockets maintained therapeutic levels and improved pocket depth more effectively than SRP alone (Pérez-Pacheco et al., 2021). Notably, performance varies by vehicle (gel/irrigant/chip), concentration, release profile, and (for any light-activated protocols) device parameters—factors that contribute to between-study variability.

#### 3.1.4 Clinical outcomes and patient benefits

Patients using curcumin adjuncts report faster resolution of gingival swelling and bleeding. Inflammatory markers diminish, and oxidative stress markers like malondialdehyde in gingival fluid are lowered, indicating reduced tissue damage and enhanced healing (Inchingolo et al., 2024). Animal studies confirm curcumin’s potential to inhibit bone resorption *via* RANKL suppression and osteoprotegerin upregulation.

Additionally, curcumin has shown promise in peri-implantitis management. Preliminary findings suggest that curcumin gels reduce peri-implant pocket depths and inflammation by targeting biofilm-induced peri-implant disease similarly to periodontitis.

Curcumin effectively supports periodontal therapy by modulating host inflammation and suppressing subgingival pathogens. It functions as a safe adjunct—either as a mouthwash to reduce gingivitis or a local agent (gel, chip, or irrigant) to boost SRP outcomes. Its lack of adverse effects and antibiotic resistance issues make it ideal for long-term maintenance in periodontal care (Zhang et al., 2022; Mohammad, 2020).

### 3.2 Dental caries and endodontic infections—antimicrobial/anti-biofilm control and root-canal disinfection

Dental caries (tooth decay) is initiated by acidogenic bacteria (notably *Streptococcus mutans* and *Lactobacilli*) that demineralize enamel. Curcumin’s antimicrobial and anti-biofilm effects lend themselves to caries prevention strategies. While fluoride remains the cornerstone of caries prevention, curcumin is being explored as a complementary agent in anticaries varnishes, fillings, and dental materials.

*In vitro*, curcumin potently inhibits *S. mutans* growth and blocks its biofilm formation on tooth-mimicking surfaces (Dai et al., 2022). As mentioned earlier, curcumin interferes with *S. mutans* enzymes (sortase, glucosyltransferases) necessary for plaque formation. One study (Dai et al., 2022) found that a curcumin-containing resin composite applied to tooth surfaces released curcumin, which significantly reduced *S. mutans* colonization and lactic acid production, compared to a control composite (Inchingolo et al., 2024). This suggests that incorporating curcumin into fissure sealants or restorative materials could impart antibacterial properties and protect adjacent enamel from secondary caries. Indeed, an innovative bio-nanocomposite of carboxymethyl starch-chitosan loaded with curcumin achieved a 91% curcumin entrapment and slow release, effectively inhibiting *S*. *mutans* and preventing biofilm formation over time. Such materials may act as drug delivery systems to high-risk sites (pits, fissures, margins of restorations) to continuously exert anticaries effects. These findings are preclinical (*in vitro*/materials science) and demonstrate feasibility rather than clinical effectiveness; no randomized clinical trials have yet shown caries-preventive benefit for curcumin-eluting dental materials.

Curcumin has also been tested as part of herbal mouthwashes or toothpastes for caries control. A study on a curcumin-based toothpaste found that when used by children over 6 months, it significantly lowered salivary *S. mutans* counts and reduced new carious lesion development, performing on par with a fluoride toothpaste (Inchingolo et al., 2024). The antimicrobial action of curcumin in the toothpaste was credited with these outcomes. Some commercially available natural toothpaste brands now include curcumin (often labeled as “turmeric extract”) for its purported plaque and caries-fighting abilities. These findings are preclinical (*in vitro*/materials science) and demonstrate feasibility rather than clinical effectiveness; no randomized clinical trials have yet shown caries-preventive benefit for curcumin-eluting dental materials. Clinical evidence for toothpaste/mouthwash formulations remains limited by small samples and short follow-up, and curcumin products should be positioned as adjuncts—not replacements—for fluoride-based prevention.

In the realm of endodontics (root canal therapy), curcumin’s antimicrobial effect has been harnessed for disinfection of infected root canals. *Enterococcus faecalis* is a resilient bacterium often found in persistent endodontic infections. Curcumin, especially in combination with light activation (antimicrobial photodynamic therapy), has reduced *E. faecalis* biofilms within root canals *in vitro* and *ex vivo* (Narayanan et al., 2020). Curcumin can be used as an irrigant solution or canal medicament; when activated with a blue light laser, it produces reactive oxygen that kills bacteria within dentinal tubules. Studies have found curcumin-PDT to be comparable to sodium hypochlorite in several *ex vivo* models in reducing bacterial load, but head-to-head clinical trials *versus* standard irrigants are lacking, and PDT protocols (photosensitizer concentration, light wavelength/fluence) vary across studies.

Another caries-related application is curcumin’s use in pit and fissure sealants combined with photoactivation (Chaturvedi, 2009). Researchers have experimented with curcumin as a photosensitizer in sealing deep pits on teeth, where shining curing light with curcumin yields a dual effect: polymerizing the sealant and simultaneously killing any residual bacteria in the fissure. This could improve the success of sealants in arresting early pit lesions. This is a proof-of-concept methodology; clinical outcomes (caries arrest/incidence) have not yet been established in randomized trials.

Moreover, curcumin’s anti-inflammatory property might be beneficial in deep carious lesions approaching the pulp (Birjandi and Sharpe, 2025). A curcumin-based lining material placed under a filling in a deep cavity could help reduce pulpal inflammation and promote healing (*via* curcumin-induced odontoblast-like cell stimulation, as some *in vitro* studies on dental pulp cells suggest). Curcumin has been noted to induce mineralization activity in stem cells of the dental pulp (Yang et al., 2023), hinting at a role in regenerative endodontics, although this is an emerging area. These signals derive primarily from cell and animal models; clinical efficacy and safety for pulpal therapy remain to be confirmed.

In summary, while fluoride and mechanical plaque control remain primary for caries management, curcumin offers an adjunctive tool. Preclinical innovations (material elution systems, anti-biofilm/PDT effects) show feasibility, whereas clinical applications (toothpaste/mouthwash adjuncts; endodontic disinfection) are promising but require larger, well-controlled trials to confirm efficacy and define protocols.

### 3.3 Recurrent aphthous stomatitis—pain reduction and accelerated healing (steroid-sparing)

Recurrent aphthous stomatitis (aphthous ulcers or “canker sores”) is a common oral mucosal condition characterized by painful, recurring ulcers on the non-keratinized oral mucosa. The exact etiology is unclear but involves local trauma, immune dysregulation, and possibly oxidative stress. Conventional management often uses topical corticosteroids (like triamcinolone acetonide) to reduce ulcer inflammation and pain. Curcumin, with its anti-inflammatory and wound-healing properties, has been investigated as a therapeutic option for RAS.

A growing body of evidence indicates that curcumin is effective in reducing the severity and accelerating the healing of aphthous ulcers. A systematic review of nine clinical trials with 469 patients found that curcumin significantly reduced ulcer size, pain intensity, and sometimes the number of ulcers, especially when applied as a gel, paste, or mouthrinse (Al-Maweri et al., 2022). In a double-blind randomized clinical trial, a 2% curcumin oral gel led to significant reductions in pain (often pain-free by day 3–4) and ulcer size compared to placebo (Deshmukh and Bagewadi, 2014). Similarly, a randomized trial comparing curcumin gel to triamcinolone gel found both produced comparable improvements by day 7, but curcumin avoided steroid-related side effects (Raman et al., 2020).

In head-to-head clinical comparisons, topical curcumin has demonstrated non-inferiority to standard therapy. Four trials directly compared topical curcumin with triamcinolone acetonide, showing that curcumin achieved similar improvements in ulcer healing time and pain relief (Gharibpour et al., 2021). One randomized study of 45 patients found average healing time to be ∼5 days in both curcumin and triamcinolone groups, which was faster than ∼7 days in the control group (Bakhshi et al., 2022). Pain scores measured by the visual analog scale also decreased significantly within 48 h of curcumin use.

Mechanistically, curcumin reduces local inflammation, oxidative stress, and immune dysregulation around ulcers. Biopsies from curcumin-treated ulcers revealed reduced pro-inflammatory cytokines and lower inflammatory cell infiltration compared to untreated controls (Al-Maweri et al., 2022). Curcumin also promotes re-epithelialization, facilitating faster ulcer closure. Evidence suggests that curcumin may downregulate T-cell activation implicated in RAS pathogenesis.

Some studies have reported that regular curcumin use may reduce recurrence frequency. Patients with chronic RAS who used curcumin gels during flare-ups experienced longer ulcer-free intervals during follow-up compared to previous patterns (Gharibpour et al., 2021). This may be due to curcumin’s immunomodulatory effects, which maintain a more quiescent oral mucosal environment.

Curcumin’s excellent safety profile makes it suitable for long-term or recurrent use-unlike steroids, which can cause mucosal thinning or oral candidiasis. Curcumin is well-tolerated, non-toxic, and lacks the stinging taste of antiseptics such as chlorhexidine. This makes it especially appealing for children and steroid-intolerant individuals.

Formulations include turmeric-based mouthrinses, curcumin orabase, and adhesive patches. In a randomized trial (Deshmukh and Bagewadi, 2014), a 5% curcumin orabase performed comparably to 0.1% triamcinolone, significantly reducing pain and lesion size within 5 days. Emerging technologies like curcumin-loaded oral patches have shown enhanced healing due to sustained release and localized protection.

Most RAS trials are small or pilot studies with short follow-up; histopathological validation is not applicable, and long-term safety or relapse-prevention data are limited.

In summary, curcumin represents a promising non-steroidal treatment for RAS. Clinical trials consistently show that curcumin reduces ulcer size, pain, and healing time with outcomes comparable to corticosteroids (Al-Maweri et al., 2022; Deshmukh and Bagewadi, 2014). Its excellent safety and tolerability make it ideal for long-term management, especially in patients with frequent recurrences or corticosteroid contraindications.

### 3.4 Oral lichen planus—symptom control and steroid-sparing potential (mixed evidence)

Oral lichen planus is a chronic immune-mediated mucosal condition, often considered a premalignant disorder, characterized by T-cell-mediated damage to basal keratinocytes and presenting with white striations, erythema, and ulcerations on the oral mucosa (Shi et al., 2024). Patients suffer from pain and burning, especially in erosive OLP. First-line therapy is usually potent topical corticosteroids or calcineurin inhibitors to control inflammation. Curcumin’s anti-inflammatory and immunomodulatory effects have made it an interesting candidate for OLP management, and several trials have explored its use either topically or systemically.

The evidence for curcumin in OLP is mixed, with some studies reporting significant improvements and others finding no difference *versus* placebo or standard treatment. For instance, a 2012 randomized controlled trial comparing 1% topical curcumin gel with triamcinolone in 50 OLP patients found significant reductions in pain and lesion size in both groups, with no significant differences between them (Mansourian et al., 2017). Similarly, Chainani-Wu et al. (2007) conducted a pilot trial using high-dose systemic curcumin (6,000 mg/day orally for 3 months) and observed marked reductions in symptom severity in the majority of patients, suggesting systemic curcumin could be beneficial for more extensive lesions.

However, recent systematic reviews have provided more nuanced perspectives. A 2020 review concluded that although curcumin reduced inflammatory cytokines and improved some clinical parameters, overall outcomes such as lesion erythema, pain, and ulcer size were not significantly different from placebo or corticosteroids (Lu et al., 2018). A 2024 meta-analysis supported this, noting significant heterogeneity among studies and variable efficacy depending on treatment duration and formulation (Shi et al., 2024). For example, short-duration (2-week) curcumin trials showed better results in pain reduction, while longer trials often found no differences between curcumin and placebo (Gupta et al., 2017).

Nonetheless, curcumin has shown non-inferiority in multiple head-to-head trials. In one split-mouth study, curcumin and triamcinolone both led to comparable improvements in lesion size and pain when applied to opposing sides of the mouth (Kia et al., 2020). In another placebo-controlled trial with systemic curcumin (2 g/day), improvement was observed in both curcumin and placebo groups, indicating either a placebo effect or insufficient dosing of curcumin (Kia et al., 2020).

Mechanistically, curcumin may reduce pathogenic immune activity in OLP through downregulation of NF-κB and cytokines such as IL-6, IL-8, and TNF-α, as shown in biopsy-based studies (Zeng et al., 2022). Histological changes such as reduced basal layer liquefactive degeneration and thinning of the lymphocytic band have also been observed following curcumin treatment (Gupta et al., 2017). Additionally, curcumin may promote apoptosis of hyperactive T cells in OLP lesions, contributing to lesion resolution.

Recent interest has focused on combination strategies. A 2021 trial using nanocurcumin gel plus 0.1% triamcinolone rinse showed that this combination enhanced mucosal healing compared to steroid alone, suggesting curcumin may act as a steroid-sparing agent (Kozuch and Hanauer, 2008). Nanocurcumin offers better mucosal penetration, which could explain the improved outcomes.

Sample sizes are modest and risk-of-bias elements vary; where biopsies were not repeated, histopathological confirmation of response is lacking, and long-term safety/durability remains insufficiently characterized.

In summary, curcumin shows promise for OLP management, particularly in mild-to-moderate cases or as an adjunct to reduce reliance on steroids. Its excellent safety profile, absence of mucosal atrophy, and tolerability support its continued investigation. While high-quality RCTs are still needed, clinicians may consider trialing 2%–3% topical curcumin (three to four times/day) in patients refractory to or intolerant of conventional therapy, with realistic expectations regarding variability in patient response.

### 3.5 OSF and leukoplakia—symptom relief, mouth-opening gains, and histologic signals

Oral submucous fibrosis (OSF) is a chronic, progressive fibrotic condition of the oral mucosa strongly associated with areca nut chewing. It is characterized by inflammation and excessive collagen deposition in the submucosa, leading to stiffening of tissues, trismus (limited mouth opening), burning sensation, and a risk of malignant transformation to oral cancer. Oral leukoplakia is a white precancerous lesion often linked to tobacco use. Both conditions involve oxidative stress and a field of molecular changes predisposing to cancer. Curcumin’s anti-inflammatory and antioxidant effects, as well as its ability to modulate fibrosis pathways, make it a logical therapeutic candidate in these potentially malignant disorders.

#### 3.5.1 Clinical improvements in OSF

Multiple studies have indicated that curcumin can ameliorate the symptoms and signs of OSF. Curcumin has been given both topically (as oral paint/gel) and systemically (oral capsules) in OSF patients. A landmark study by Hazarey et al. (2015) treated OSF patients with curcumin supplements for 3 months and observed significant improvements in mouth opening and reductions in oral burning sensation compared to baseline and to a placebo group. Patients taking curcumin were able to open their mouths wider by a few millimeters on average, which is meaningful in a condition where the fibrosis severely limits jaw movement. Another RCT by Das et al. compared curcumin capsule plus topical turmeric oil vs. standard therapy (intralesional steroid injections) and found that the curcumin group had comparable improvement in mouth opening and far better improvement in subjective symptoms (like burning and intolerance to spicy foods) without the injection-related discomfort (Hazarey et al., 2015).

In a controlled trial, curcumin (both systemic and topical) led to a significant reduction in OSF symptoms: burning sensation was markedly reduced, and patients reported better flexibility of the tongue, lips, and cheeks. Objective measurements showed an increase in maximal mouth opening (often by 5–8 mm) and improved cheek flexibility (implying reduction of fibrotic bands) in the curcumin-treated group (Inchingolo et al., 2024). These improvements are likely tied to curcumin’s inhibition of key fibrogenic drivers. Biochemical analyses revealed that after curcumin therapy, there was a reduced expression of fibrosis-related proteins such as TGF-β, p53, and iNOS in OSF tissues. TGF-β is a master cytokine promoting fibroblast activation and collagen synthesis; iNOS and aberrant p53 are often upregulated in OSF mucosa and considered biomarkers of carcinogenic progression. Curcumin’s ability to lower these indicates a reversal of pathological molecular changes, aligning with clinical softening of tissues.

A recent randomized trial by Adhikari et al. (2022) provided high-quality evidence supporting curcumin’s role in OSF. They investigated curcumin in combination with intralesional steroid and hyaluronidase injections vs. injections alone in OSF patients. The addition of curcumin significantly enhanced outcomes: the combination group had greater improvement in mouth opening and tongue protrusion, as well as more relief from fibrotic tightness, than the group receiving steroid + hyaluronidase without curcumin. The authors concluded that curcumin with intralesional therapy is *“efficacious in the treatment of OSF”*, potentially by synergistically reducing inflammation and fibrosis. Moreover, curcumin has been compared to systemic antioxidant supplements like lycopene in OSF; some studies found curcumin equally or more effective than lycopene in improving symptoms, which underscores curcumin’s strong antioxidant impact in the oral mucosa.

#### 3.5.2 Mechanistic insights

Curcumin’s benefits in OSF can be attributed to multiple mechanisms: it inhibits fibroblast proliferation and collagen deposition in the mucosa, partly through downregulating TGF-β signaling and fibrogenic gene expression (Inchingolo et al., 2024). It also reduces inflammatory cytokines (like IL-6, IL-8) in the submucosal tissues, thereby mitigating the chronic inflammation that stimulates fibrosis. Additionally, curcumin has been shown to induce collagenase activity, which may help break down existing collagen bundles. Its antioxidant effect likely protects the tissues from areca nut-induced oxidative damage, which is known to contribute to fibroblast activation. One study found curcumin therapy elevated salivary levels of vitamins C and E in OSF patients while lowering malondialdehyde (MDA), indicating a restoration of antioxidant capacity in the oral environment. This could help halt the progression of OSF and even possibly regress some of the premalignant changes by preventing oxidative DNA damage.

#### 3.5.3 Leukoplakia and other precancerous lesions

For oral leukoplakia, evidence for curcumin is less abundant, but a few studies suggest a potential benefit. A randomized double-blind placebo-controlled phase IIB trial treated patients with oral leukoplakia using curcumin (3.6 g daily for 6 months) and noted a significant and durable clinical response, including reduction in lesion size and histopathological improvement in terms of reduced dysplasia in some cases (Kuriakose et al., 2016). A pilot study suggests curcumin lozenges may benefit leukoplakia patients, but the small sample size limits definitive conclusions (Khare et al., 2024). While leukoplakia lesions do not cause functional symptoms, the rationale is that curcumin might reduce the molecular drivers of dysplasia in these lesions. There are reports of leukoplakia patches showing partial regression or not progressing to worse dysplasia over a year of curcumin usage, but robust evidence is still needed. Evidence derives largely from small or pilot trials; repeat biopsy or standardized histopathological regression is infrequently documented, and there are no data demonstrating prevention of malignant transformation with curcumin to date.

#### 3.5.4 Preventing malignant transformation

A key goal in managing OSF and leukoplakia is preventing progression to oral squamous cell carcinoma. Curcumin’s anti-cancer effects are highly relevant here. In OSF patients treated with curcumin, researchers observed an increase in protective factors (like reduced mutagenic p53 accumulation) and a decrease in angiogenic and invasion markers (VEGF, MMP-9) (Shao et al., 2024). Curcumin also induces apoptosis in dysplastic or premalignant cells and enhances immune surveillance. For instance, curcumin therapy in patients with oral precancer was associated with an increase in cytotoxic CD8+T-cells in lesions and a decrease in immunosuppressive T-reg cells, potentially heightening the body’s ability to eliminate atypical cells (Shao et al., 2024). These immune shifts, along with activation of autophagy in damaged cells, could underlie the lower observed malignant transformation rates in some curcumin-treated cohorts (though long-term data are limited). Many OSF studies are single-center with limited numbers and short observation; histopathological endpoints are inconsistently reported, and longer-term safety and progression outcomes are absent.

Overall, curcumin appears to be a valuable adjunct in OSF management: it improves clinical function (mouth opening, pliability of tissues) and provides symptomatic relief, while also potentially reversing or halting some pathological changes. In leukoplakia, it may help by reducing oxidative stress and dysplasia, though evidence of lesion resolution is not yet conclusive. Given the lack of definitive treatments for OSF (besides surgery in advanced cases), curcumin’s safety and multifactorial benefits make it an attractive option to use early in the disease. It is often recommended along with habit cessation (areca, tobacco) and other antioxidants. Future large-scale trials and perhaps combination approaches (e.g., curcumin with other antifibrotic agents) will further elucidate its role, but current data justify incorporating curcumin into the therapeutic regimen for patients with OSF and possibly those with leukoplakia who are at risk for progression.

### 3.6 Oral candidiasis—antifungal efficacy and photodynamic synergy

Oral candidiasis, commonly caused by *Candida albicans*, is an opportunistic fungal infection of the oral mucosa. It frequently affects infants, denture wearers, or individuals who are immunocompromised or using corticosteroids. Symptoms include white plaques, redness, soreness,or burning of the mucosa. Standard treatment uses topical or systemic antifungals (nystatin, clotrimazole, fluconazole).

Curcumin’s antifungal activity has prompted research into its use for oral candidiasis, particularly given the rise of antifungal resistance and the need for alternative therapies. Preclinical research has clearly shown that curcumin is active against *Candida* species, including *C. albicans* and *C. glabrata*. *In vitro*, curcumin inhibits the growth of *C. albicans* at low concentrations and can kill the yeast cells at slightly higher concentrations. Notably, curcumin had inhibitory effects on *C. albicans* strains that were resistant to nystatin and fluconazole, with MIC values in the range of ∼8–32 μg/mL (Kubizna et al., 2024). The mechanisms include disrupting fungal cell membranes, generating intracellular ROS, and inhibiting the yeast-to-hyphae transition (Al-Ghamdi et al., 2023).

Clinically, curcumin has been applied in a few pilot studies for oral candidiasis, often in the form of mouthwash or gel. In denture-related stomatitis, patients using turmeric mouthrinse or curcumin gel experienced reduced mucosal inflammation and lower *Candida* counts on denture surfaces. In a randomized trial, curcumin mouthrinse performed comparably to chlorhexidine in reducing *Candida* colony counts, but with fewer side effects (Labban et al., 2021).

A breakthrough came with the use of curcumin-mediated photodynamic therapy (PDT) for oral thrush. Curcumin acts as a photosensitizer, producing ROS upon light activation that damages fungal cells. In a mouse model of oral candidiasis, curcumin-PDT significantly reduced fungal load and inflammation, comparable to nystatin treatment (Sakima et al., 2075). Human studies have also demonstrated PDT efficacy in resistant oropharyngeal candidiasis, especially in HIV-positive individuals (Shahi Ardakani et al., 2024).

For denture disinfection, curcumin has been evaluated in soaking solutions, showing a reduction in multispecies biofilms and fungal burden on acrylic surfaces. Nanoparticle-encapsulated curcumin incorporated into denture liners is under investigation as a slow-release antifungal barrier (Casu et al., 2022).

In terms of patient outcomes, curcumin treatment often leads to quick symptom relief (e.g., less burning, soreness), likely due to both antifungal and anti-inflammatory effects. In angular cheilitis, topical curcumin ointment reduced fissuring and erythema within 1 week of application.

To summarize, curcumin offers a safe, effective, and well-tolerated antifungal strategy for oral candidiasis. It shows promise, especially in drug-resistant strains and when used with photodynamic therapy (Hu et al., 2023). In some cases, curcumin can even restore fluconazole susceptibility in resistant *Candida* strains. Further trials are needed to define optimal formulations, dosages, and long-term efficacy, but the current evidence supports its adjunctive use in oral fungal infections.

### 3.7 Oral mucositis (RT/CT)—prophylaxis and symptom mitigation

Oral mucositis is a debilitating side effect of cancer therapy, particularly in patients receiving head and neck radiation or high-dose chemotherapy (e.g., for hematologic cancers). It manifests as painful inflammation and ulceration of the oral mucosa, leading to difficulty eating, swallowing, and increased risk of infection. There is no definitive treatment, and management is mainly supportive (mouth rinses, analgesics, coating agents). Curcumin has gained attention for preventing or alleviating oral mucositis due to its anti-inflammatory and wound-healing properties, and a number of clinical studies have been conducted. Recent randomized clinical evidence demonstrates symptom and severity benefits. In a 2023 single-blind RCT in head-and-neck RT patients (n = 37 completers), both a 0.1% curcumin mouthwash and a curcumin nanocapsule reduced WHO mucositis grade and pain scores *versus* placebo during weeks 1–3 (Ramezani et al., 2023). Two recent meta-analyses further support reductions in incidence/severity and pain, while emphasizing heterogeneity in formulations and dosing and the need for larger trials (Wu et al., 2024; Elad et al., 2013).

Clinical trials of curcumin for oral mucositis are generally small to moderate in size, with heterogeneous dosing schedules and delivery systems. Importantly, studies distinguish prophylactic (preventive) use (initiated before or at the start of RT/CT to reduce incidence/severity) from therapeutic use (to treat established mucositis). While signals are favorable in both contexts, effect sizes differ by formulation and timing, and larger, multi-center RCTs with standardized outcomes are needed.

#### 3.7.1 Preventive use and outcomes

Several randomized controlled trials have tested curcumin mouthwash or gel as a prophylactic agent in patients undergoing radiotherapy for head and neck cancer. The typical protocol involves patients gargling with a curcumin solution (often turmeric extract in water or nanocurcumin formulations for better solubility) before and after each radiation session. A consistent finding is that curcumin use delays the onset of mucositis and reduces its severity compared to control. For instance, Shah et al. (2020) conducted a triple-blind, pilot randomized controlled trial with 74 head and neck cancer patients receiving radiotherapy. They found that patients using 0.1% curcumin mouthwash experienced a significant delay in the onset of oral mucositis and a reduction in severity compared to those using benzydamine mouthwash. Specifically, the onset of mucositis was delayed by approximately 2 weeks in the curcumin group, and fewer patients developed severe (Grade 3) mucositis. Additionally, curcumin-gargling patients had fewer breaks in radiotherapy (since mucositis often forces treatment interruptions) and experienced less weight loss, reflecting better oral intake.

In chemotherapy-induced mucositis, similar benefits have been observed. A randomized, placebo-controlled study evaluated the effect of bio-enhanced turmeric formulation (BCM-95®) in oral cancer patients undergoing chemoradiotherapy. The study concluded that BCM-95® significantly reduced severe oral mucositis, dysphagia, oral pain, and dermatitis in these patients (Soni et al., 2022). Notably, none of the curcumin rinse users had to discontinue cancer therapy due to mucositis, whereas some control patients did. Another study by Shah et al. demonstrated that curcumin mouthwash was effective in reducing the incidence and severity of radiation-induced oral mucositis among head and neck cancer patients.

#### 3.7.2 Therapeutic use in established mucositis

Curcumin has also been tried as a treatment for existing mucositis lesions. A study by Fardad et al. (2023) compared three interventions in cancer patients with chemotherapy-induced mucositis: chlorhexidine mouthwash, standard mucositis care (benzydamine) spray, and a curcumin gel applied to lesions. Results showed that all three reduced mucositis severity, but curcumin gel achieved faster and more complete healing with fewer side effects (Dipalma et al., 2024). This indicates curcumin not only prevents but can actively promote healing of mucosal ulcers, likely through enhancing re-epithelialization and granulation tissue formation. Patients using curcumin gel reported a soothing effect and quicker pain relief, presumably as the gel formed a protective layer and curcumin reduced inflammation underneath.

Mechanistically, curcumin combats mucositis by downregulating the inflammatory pathways triggered by radiation/chemo—for example, it inhibits NF-κB activation in oral mucosal cells, thereby reducing production of mucositis-driving cytokines like TNF-α, IL-6, and reducing COX-2-mediated mucosal injury. A study (Chen et al., 2024) noted that curcumin users had lower salivary levels of IL-6 and IL-1β during therapy than controls. Curcumin also upregulates local growth factors like EGF that help mucosal healing. In one trial, curcumin-treated mucositis patients demonstrated higher saliva EGF levels and correspondingly better healing, implicating curcumin in stimulating regenerative pathways. Furthermore, curcumin’s antioxidant action protects oral mucosal cells from therapy-induced oxidative stress, and its anti-microbial effect may help prevent secondary infections of ulcerated mucosa (Amatto et al., 2025).

An interesting combination approach is using curcumin with light therapy (photobiomodulation). A study (Ribeiro da Silva et al., 2018) combined low-level laser therapy with curcumin-mediated photodynamic therapy for treating mucositis and found that adding curcumin-PDT led to faster reduction in ulcer size than laser alone. The curcumin group showed earlier onset of healing, suggesting synergy between curcumin’s effects and laser’s stimulation of cell proliferation (de Cássia Dias Viana Andrade et al., 2022).

Taken together, the evidence strongly supports curcumin’s role in both preventing and treating oral mucositis in cancer patients. Multiple systematic reviews conclude that curcumin is among the most promising natural agents for mucositis management, showing consistent reductions in severity across trials. Patients benefit by experiencing less pain (thus requiring fewer opioid analgesics), maintaining better oral intake, and having an overall improved quality of life during cancer therapy. The simplicity of using a curcumin rinse or paste and its low cost further add to its appeal.

It is worth noting that in none of the mucositis trials did curcumin cause adverse effects—patients did not develop taste issues or mucosal staining; in fact, curcumin’s pleasant taste compared to medicinal rinses can improve compliance. As a result, some oncology centers have started incorporating curcumin mouthwashes into their standard preventive oral care protocols for patients starting head-neck radiotherapy or bone marrow transplant conditioning.

### 3.8 Oral squamous cell carcinoma—biologic modulation and quality-of-life signals (early clinical evidence)

Oral squamous cell carcinoma (OSCC) is the most common oral malignancy, often arising from precancerous lesions or in the context of risk factors like tobacco, alcohol, or HPV infection. Treatment involves surgery, radiation, and chemotherapy, but survival rates remain modest, and new therapeutic strategies are needed. Curcumin has garnered interest in oncology due to its ability to modulate cancer cell growth and metastasis pathways, induce apoptosis, and enhance the efficacy of conventional treatments. In the context of oral cancer, curcumin has been studied in laboratory models, and early-phase clinical trials have explored its effects either as a chemopreventive or as an adjuvant to therapy. Translational data in patients suggest biological activity at the tumor–host interface. In a randomized, placebo-controlled phase-1 ‘window’ trial, a curcumin-containing botanical (APG-157) achieved measurable systemic absorption and reduced salivary pro-inflammatory cytokines (IL-1β, IL-6, IL-8) and tumor-associated microbes in OSCC patients (Basak et al., 2020). While early-phase outcomes are promising, major cancer agencies still judge evidence as insufficient for routine oncologic treatment and call for larger, well-controlled trials (PDQ Integrative et al., 2024).

At present, curcumin should be regarded as an adjunct or investigational add-on rather than a primary oncologic therapy; standard management continues to rely on surgery, radiotherapy, and systemic therapy, with curcumin explored primarily for biomarker modulation and symptom control.

In OSCC, evidence is largely preclinical or early-phase. Existing randomized ‘window’ trials are small and focus on biomarker and microbiome modulation; there is no evidence yet for survival or local control benefit. Accordingly, curcumin should not be represented as a therapeutic oncologic treatment outside clinical trials. Its potential preventive role pertains mainly to premalignant conditions (e.g., leukoplakia, oral submucous fibrosis), where symptomatic and functional improvements have been reported, but durable cancer-preventive efficacy remains uncertain.

#### 3.8.1 Anti-cancer effects in OSCC models

*In vitro* studies on OSCC cell lines consistently show that curcumin inhibits cancer cell proliferation and induces apoptosis. Curcumin causes a G2/M cell cycle arrest in oral cancer cells and triggers the intrinsic apoptosis pathway by increasing pro-apoptotic proteins (Bax, cleaved caspases) and decreasing anti-apoptotic proteins (Bcl-2) (Joshi et al., 2021). Curcumin also interferes with multiple signaling pathways that are often dysregulated in OSCC. For example, it downregulates NF-κB and STAT3 signaling in oral cancer cells, thereby reducing expression of genes that promote tumor survival and inflammation (like cyclin D1, COX-2, IL-6) (Burche et al., 2023). It also suppresses angiogenesis by inhibiting VEGF production and inhibits invasion and metastasis by affecting matrix metalloproteinases (MMP-2, MMP-9) and epithelial-mesenchymal transition (EMT) markers (Singh et al., 2023; Ohnishi et al., 2020). An important finding is that curcumin reduced the expression of EMT drivers such as Snail and Twist in OSCC cells and upregulated E-cadherin (which is typically lost during EMT). This indicates curcumin can help maintain epithelial characteristics and reduce the invasive potential of oral cancer cells. Concurrently, curcumin can boost tumor suppressor pathways; for instance, it has been shown to increase the activity of p53 (when wild-type p53 is present) in OSCC, enhancing the cell’s ability to undergo apoptosis in response to DNA damage (Schiavoni et al., 2025; Kumar and Jha, 2023).

In animal models of oral cancer (such as chemically induced tongue tumors in hamsters or mice), dietary or topical curcumin has resulted in smaller and fewer tumors, as well as histologically less aggressive lesions, compared to controls (Budi and Farhood, 2023). These chemopreventive effects align with curcumin’s activity in precancerous conditions like OSF/leukoplakia noted earlier. Curcumin-fed animals show lower rates of conversion of dysplasia to carcinoma. Moreover, in xenograft models (human OSCC tumors implanted in mice), curcumin treatment has been shown to inhibit tumor growth and reduce lung metastases (Davoodvandi et al., 2021). Mechanistically, tumors from curcumin-treated models display reduced microvessel density (due to VEGF inhibition) and increased apoptotic cell density.

#### 3.8.2 Clinical trials and translational studies

Although no large clinical trial has yet proven curcumin as a definitive treatment for oral cancer, early-phase trials demonstrate its potential as an adjunct to existing treatments:

A Phase I clinical trial (Trošelj et al., 2020) investigated a formulation called APG-157 (curcumin in a lozenge form with enhanced bioavailability) in patients with oral/oropharyngeal cancer. The results showed that APG-157 achieved measurable levels of curcuminoids in oral tissues and blood, confirming systemic absorption from oral delivery. More importantly, treatment with APG-157 led to a reduction in pro-inflammatory cytokines IL-1β, IL-6, and IL-8 in the saliva of these cancer patients (Gugnacki et al., 2021). Chronic elevation of these cytokines in the tumor microenvironment is associated with cancer progression and cachexia; curcumin’s ability to lower them suggests it can favorably modulate the tumor milieu. Additionally, the salivary microbiome in these cancer patients shifted–APG-157 usage significantly reduced the abundance of certain tumor-associated bacterial species (like those from phylum Bacteroidetes) that have been linked to oral cancer inflammation. The treatment was well tolerated with no dose-limiting toxicities. The study concluded that curcumin lozenges could be a useful neoadjuvant or adjuvant by altering the tumor microenvironment and possibly enhancing the response to immunotherapy (Elmi et al., 2024; Bernitsa et al., 2023).

A Phase II trial (ongoing or recently completed) is testing APG-157 in combination with a checkpoint inhibitor (immunotherapy) in oral cancer, based on preclinical suggestions that curcumin may improve anti-tumor immunity (Xiang et al., 2019). Curcumin’s observed effects of increasing CD8^+^ T cells and decreasing immunosuppressive cells in the tumor microenvironment (Conti et al., 2024) support this strategy. If curcumin can reduce myeloid-derived suppressor cells and T-regs in human tumors as it did in animal studies, it might amplify the efficacy of immunotherapies.

Topical curcumin application on oral tumors has also been attempted in case reports. In one report, a patient with multiple small superficial tongue carcinomas applied a curcumin oral paste daily (while awaiting surgery) and was found to have no progression in lesion size over 8 weeks, with some areas showing histologic regression to lower-grade dysplasia. While anecdotal, it hints at curcumin’s direct local action on tumor cells.

#### 3.8.3 Curcumin as a chemosensitizer or radiosensitizer

Preclinical studies indicated curcumin can make cancer cells more sensitive to chemotherapy (e.g., by inhibiting NF-κB, which often confers chemoresistance). A small pilot study in head-neck cancer patients combined curcumin with standard chemoradiation and noted improved responses in the curcumin group (higher rate of complete tumor regression) compared to a matched group on chemoradiation alone, though patient numbers were limited. Curcumin’s protection of normal cells from radiation (reducing mucositis) while not protecting tumor cells could create a therapeutic window where overall outcomes improve.

#### 3.8.4 Preventive use in high-risk individuals

For those with extensive precancerous lesions or a history of treated oral cancer, curcumin supplements have been proposed as a chemopreventive to prevent second primary tumors or recurrences. While conclusive data are pending, this is supported by curcumin’s ability to target dysplastic cells and modulate pathways of carcinogenesis (Marino et al., 2023; Crooker et al., 2018).

Taken together, current evidence does not demonstrate survival or locoregional control benefit for curcumin in OSCC; larger, adequately powered randomized trials with standardized formulations and clinically meaningful endpoints are needed before any change in practice.

In summary, curcumin exhibits multi-pronged anti-cancer effects in oral oncology: it can directly inhibit tumor cells, reduce tumor-promoting inflammation, suppress angiogenesis and invasion, and potentially enhance immune attack on the tumor. Early clinical research shows curcumin can be delivered effectively to the oral tumor site (e.g., *via* lozenges) and favorably alters biomarkers. While curcumin is not a stand-alone cure for established oral cancer, it holds promise as part of an integrated approach–for example, as a neoadjuvant to shrink tumors or as an adjunct to reduce metastasis and recurrence risk. Large-scale clinical trials are needed to verify its impact on survival or tumor control. If proven, curcumin or its analogues could become a component of oral cancer chemoprevention regimens or adjunctive therapy, offering a low-toxicity means to improve patient outcomes in this disease.

## 4 Delivery systems for oral application—improving bioavailability and mucosal retention

A major hurdle for curcumin’s clinical use is its poor solubility in water, rapid metabolism, and low bioavailability when ingested. To maximize curcumin’s benefits specifically in the oral cavity, various delivery systems and formulations have been developed and tested. These aim to increase the local concentration of curcumin at oral disease sites, prolong its retention time on mucosal surfaces, or enhance its absorption into oral tissues.

### 4.1 Mouthwashes and rinses

The simplest formulation is a curcumin mouthwash. Typically, a turmeric extract or curcumin powder is dissolved or suspended in water (often with a small amount of ethanol or a solubilizing agent) to create a rinse. Patients are instructed to swish for 1–2 min to allow contact with all oral surfaces. Curcumin mouthwashes of 0.1%–1% concentration have been widely used in trials for gingivitis, mucositis, *etc.*, with positive outcomes as discussed. The advantage of a rinse is ease of use and the ability to reach all corners of the mouth. To improve curcumin’s solubility in rinses, formulations may include adjuvants like piperine (from black pepper, which inhibits curcumin metabolism and increases uptake) or use nanomicelle technology. One study utilized a nanomicellar curcumin mouthwash (SinaCurcumin®) where curcumin is encapsulated by surfactant micelles, making it water-soluble (Inchingolo et al., 2024). This formulation significantly delayed and reduced chemotherapy-induced mucositis, with no reported difficulties in use. Nanomicelles protect curcumin from precipitation and enzymatic degradation in saliva, thereby delivering more active compound to tissues (Rezaei-Tazangi et al., 2023).

### 4.2 Oral gels and pastes

Curcumin gels (typically a hydrogel base like carbopol or xanthan gum, loaded with curcumin) are useful for adhesive application on specific lesions (e.g., aphthous ulcers, OLP patches, perioperative wounds). Gels can ensure prolonged contact time. A 2% curcumin gel has been a common choice, often applied 3–4 times daily. The gel form has shown high efficacy in conditions like oral mucositis and OLP (Pan-On et al., 2022), likely because it forms a protective layer and keeps curcumin concentrated at the lesion site. Some gels combine curcumin with other ingredients; for example, curcumin has been formulated in Orabase (an adhesive paste often used for steroid application) to treat ulcers–the orabase ensures the curcumin is not quickly washed away by saliva.

### 4.3 Mucoadhesive patches and films

To further prolong exposure, researchers have developed curcumin mucoadhesive patches. These are small disks or strips (often made of polymers like hydroxypropyl cellulose or chitosan) that can stick to the oral mucosa and slowly release curcumin. A mucoadhesive film containing curcumin was tested for aphthous ulcers–patients applied the patch to the ulcer and left it in place, where it dissolved over ∼30 min, delivering curcumin into the ulcer site. This method was found to significantly reduce ulcer pain within hours and speed up healing, with the convenience of once or twice daily application rather than multiple gel applications (Gopalakrishna et al., 2023). Similarly, for OLP lesions on the buccal mucosa, curcumin films provided continuous drug delivery, and patients found them easy to use without interfering with speech or eating (as they adhere until dissolved).

### 4.4 Nanoparticle and liposomal formulations

Nanotechnology has been employed to enhance curcumin delivery. Curcumin can be encapsulated in nanoparticles (e.g., polymeric nanoparticles, solid lipid nanoparticles, or nanoemulsions) that not only improve solubility and stability but can also facilitate uptake into tissues. One example is nanocurcumin–curcumin loaded into polymer nanoparticles of ∼50–100 nm. In a trial for gingivitis, an oral nanocurcumin suspension led to greater reductions in the gingival index and bleeding on probing than a traditional curcumin prep, likely due to better penetration into the gingival crevices. Nanoparticles can also allow curcumin to penetrate biofilms more effectively, enhancing its antimicrobial action. Liposomal curcumin (curcumin enclosed in phospholipid vesicles) is another strategy; the liposomes can fuse with bacterial membranes to deliver curcumin directly where needed. Some mouthrinse formulations incorporate liposomal curcumin for improved shelf stability and mucosal absorption.

### 4.5 Local drug delivery devices for periodontitis

In periodontal pockets, maintaining drug presence is challenging due to saliva flow. Thus, intra-pocket devices have been crafted. These include curcumin strips or fibers that are inserted into deep pockets after SRP, where they slowly release curcumin over several days. A study designed a biodegradable film with curcumin (as mentioned earlier) and found it successfully released curcumin in a sustained manner in the pocket and improved periodontal outcomes. Chitosan, being mucoadhesive and biodegradable, is often used to make such films. A variant is curcumin *in situ* gels that are liquid when injected into a pocket and then solidify into a gel that adheres to the pocket walls, releasing curcumin over a week. These targeted delivery systems ensure high curcumin concentration directly at the diseased site for extended periods, which is ideal for chronic periodontitis management (Hegde et al., 2023).

### 4.6 Toothpastes and chewing gums

Curcumin has been included in some herbal toothpastes aimed at reducing plaque and gingivitis. While the contact time during brushing is short, daily use does impart some cumulative effect. Curcumin toothpastes have shown the ability to reduce oral microbial counts and inflammatory markers in the gingival crevicular fluid. Chewing gum with curcumin is an innovative concept studied in a trial for oral cancer patients–a curcumin-infused gum was chewed to deliver curcumin to oral tissues systematically. Chewing the gum released curcumin that got absorbed through the oral mucosa and led to measurable decreases in serum inflammatory markers like CXCL1 and TNF-α. This suggests a gum could be a convenient delivery mode for curcumin as a chemopreventive in populations at risk of oral or systemic disease (Hao et al., 2025).

### 4.7 Enhancers of bioavailability

Combining curcumin with bioenhancers is another strategy. Piperine is most well-known; it can increase curcumin’s bioavailability by 20-fold by inhibiting glucuronidation in the liver. Some oral supplements pair curcumin with piperine for systemic absorption. For example, patients with OSF took curcumin capsules containing piperine and saw a good clinical response, presumably because enough curcumin reached the oral tissues systemically (Mohajeri et al., 2021). Other enhancers include using curcumin in oils or lipid bases (since curcumin is lipophilic). Curcumin dissolved in coconut oil or olive oil has been used as an oil-pulling or swishing therapy in anecdotal reports for oral hygiene improvement. Indeed, curcumin’s absorption is improved when taken with oils (Harwansh et al., 2023), a fact noted in traditional uses (like mixing turmeric in ghee or milk).

To address stability, some delivery forms use curcumin analogues or derivatives with better pharmacokinetics. While not exactly “curcumin,” these modified compounds (e.g., EF24, a curcumin analog) might be included in future oral care products if they prove more effective. The characteristics of major curcumin delivery systems tailored for oral application are summarized in Table 2.

**TABLE 2:** Comparison of curcumin delivery systems designed for oral disease management. The table highlights formulation types, carrier systems, advantages, and specific use cases supported by preclinical or clinical evidence. Data derived from PubMed-indexed trials and reviews published in high-impact journals.

Mechanism	Target pathway/molecule	Disease relevance	Evidence type	References
Anti-inflammatory	NF-κB, IL-1β, IL-6, TNF-α	Periodontitis, Mucositis, OLP	*In vitro*, Clinical	Inchingolo F et al. Biochem Pharmacol. 2024 May 28; 13(6):660. (Inchingolo et al., 2024)
Antioxidant	Nrf2, HO-1, SOD	OSF, Gingivitis, Cancer	*In vitro*, Animal	Sivani BM et al. Metabolites. 2022 July 12; 12(7):639. (Sivani et al., 2022)
Pro-apoptotic	Bax↑, Bcl-2↓, Caspase-3↑	OSCC, Leukoplakia	Cell line, Animal	Djaldetti M. Oncol Res. 2024 August 23; 32(9):1389–1399 (Djaldetti, 2024)
Anti-angiogenic	VEGF↓, HIF-1α↓	Oral Cancer, OSF	Animal, Xenograft	Sudhesh Dev S et al.Front Pharmacol. 2021 November 15; 12:772510. (Sudhesh Dev et al., 2021)
Anti-microbial	Biofilm Disruption, S. mutans	Gingivitis, Caries	*In vitro*	McCubrey JA et al. Aging (Albany NY). 2017 June 12; 9(6):1477–1536. (McCubrey et al., 2017)
EMT inhibition	Snail↓, Twist↓, E-cadherin↑	OSCC (anti-metastatic)	Cell line	Hao M et al. Front Pharmacol. 2025; 16:1509045. (Hao et al., 2025)
Immunomodulation	↑CD8+ T cells, ↓T-regs	Oral Cancer (immune checkpoint synergy)	Animal, Phase I	Antonangeli F et al. Front Immunol. 2020 November 25; 11:584626. (Antonangeli et al., 2020)
Inhibits pro-inflammatory gene transcription	NF-κB	Periodontitis, Gingivitis	*In vitro*	Aggarwal BB et al.Adv Exp Med Biol. 2007; 595:1–75. (Aggarwal et al., 2007)
Blocks new vessel formation, impairs tumor supply	VEGF	Cancer Angiogenesis	*In vitro*, Cell line	Gupta SC et al. Cancer Metastasis Rev. 2018; 37(2–3):259–277. (Gupta et al., 2010)

Regulatory and formulation considerations—From a regulatory perspective, curcumin is not approved by the U.S. Food and Drug Administration (FDA) to treat any disease; most U.S./EU products are regulated as dietary supplements or cosmetics/oral-care preparations, not as drugs. Curcumin and turmeric oleoresin are permitted in foods (e.g., as color additives/GRAS), but these listings do not confer therapeutic approval. Several curcumin-containing oral-care gels are commercially available in India under Ayurvedic/OTC frameworks (e.g., Curenext™ curcuma oral gel), while a curcumin-containing botanical lozenge (APG-157) has shown biologic activity in a randomized Phase 1 trial in oral cancer and currently holds FDA Fast Track designation in head-and-neck cancer (Basak et al., 2020); however, no curcumin oral product is FDA-approved for disease treatment (PDQ Integrative et al., 2024). Formulation challenges that constrain translation include poor aqueous solubility, pH/photochemical instability, rapid metabolism, and short mucosal residence times. These issues motivate the use of mucoadhesive gels/films, nanomicelles/phytosomes/cyclodextrins, and photosensitizer formulations for aPDT to enhance solubility, retention, and local bioavailability. Standardization is also critical: curcuminoid composition (curcumin/demethoxycurcumin/bisdemethoxycurcumin ratios), excipient profiles, and release characteristics vary widely across products, complicating dose equivalence and trial comparability. Future development should predefine the regulatory route (drug vs. device vs. supplement), adopt quality-by-design and cGMP standards, and include stability/compatibility data and device–drug integration (for light-activated therapies) to support scalable, regulator-ready formulations.

In conclusion, significant progress has been made in developing tailored curcumin delivery systems for oral use. These range from simple mouthwashes to sophisticated nanocarriers and mucoadhesive devices. The goal is to maximize curcumin’s localized effect while minimizing the need for very high systemic doses. As research continues, we may see curcumin incorporated into everyday dental products (mouthwashes, dentifrices) and also into prescription devices for periodontal therapy or mucosal disease management. The array of formulations ensures that curcumin’s versatility can be practically harnessed in different clinical scenarios, improving patient compliance and outcomes.

## 5 Clinical trials and evidence synthesis

The therapeutic potential of curcumin in oral health has been assessed in numerous clinical studies over the past two decades. These trials, varying in size and design, collectively provide a robust evidence base supporting curcumin’s efficacy and safety for various oral conditions. Here, we synthesize findings from key clinical trials and systematic reviews (2000–2025) to present a consolidated view of the current evidence, A summary of representative clinical studies evaluating curcumin’s efficacy across different oral conditions is presented in Table 3.

**TABLE 3:** Summary of clinical trials evaluating curcumin formulations in various oral diseases.

Disease	Study type	Delivery method	Dose/formulation	Age (Years)	Key outcomes	References
Periodontitis	RCT (n = 60)	Gel (2% curcumin)	Twice daily for 14 days	25–55	↓ Gingival Index, Probing Depth vs. SRP alone	Anuradha BR et al. J Indian Soc Periodontol. 2015; 19(3):302. (Anuradha et al., 2015)
Oral Mucositis (Radiotherapy)	RCT (n = 40)	Mouthwash (0.1%)	3×/day during RT	30–65	↓Mucositis grade, ↓ Pain scores, ↑ Oral intake	Chakraborty, S et al. Br J Radiol. 2015; Apr; 88(1048):20140795. (Chakraborty et al., 2015)
Recurrent Aphthous Ulcer	RCT (n = 50)	Gel (5%)	Topical application 3×/day	18–45	↓ Healing time, ↓ Pain comparable to triamcinolone gel	Chainani-Wu N et al. J Altern Complement Med. 2007; 13 (1):123–9. (Chainani-Wu et al., 2007)
Oral Lichen Planus	RCT (n = 40)	Capsules (500 mg)	Twice daily for 3 months	35–60	↓ Burning sensation, ↓ Lesion size, comparable to steroids	Singh AK et al. J Maxillofac Surg.2023 January-Apr; 14(1):9–15. (Singh et al., 2023)
Oral Submucous Fibrosis	RCT (n = 100)	Tablets + Topical	600 mg/day + gel	20–50	↑ Mouth opening, ↓ Fibrosis stage	Rai, Arpita et al. J Stomatol Oral Maxillofac Surg.2023 June; 124(3):101423. (Rai et al., 2023)
Oral Cancer (APG-157 trial)	Phase I	Lozenge (APG-157)	100 mg/day	40–70	↓ IL-1β, IL-6, IL-8 in saliva; altered microbiome	Basak SK et al. Cancer. 2020; 126(Suppl 10):2056–69. (Basak et al., 2020)

Abbreviations: GI, gingival index; PI, plaque index; PPD, probing pocket depth; CAL, clinical attachment level; OM, oral mucositis; VAS, visual analog scale. All studies listed were indexed in PubMed and published in peer-reviewed journals.

### 5.1 Periodontal disease trials

At least 18 randomized controlled trials (RCTs) have evaluated curcumin as an adjunct in periodontal therapy. As summarized by a 2022 meta-analysis, these trials (totaling ∼846 patients) consistently show that adjunctive curcumin (gel, irrigation, or mouthwash) with standard scaling and root planing yields greater reductions in the gingival index, bleeding index, and pocket depth than scaling and root planing alone (Afrasiabi et al., 2022). No serious adverse effects were reported, and curcumin was often noted to improve patient-reported outcomes (less gum soreness after deep cleaning, *etc.*). These findings establish curcumin as an effective adjunct, on par with other adjuncts like chlorhexidine or antibiotics, but with fewer downsides (no staining or resistance issues). While most periodontal trials are short-term (4–12 weeks), a few longer studies indicate that a curcumin adjunct can sustain periodontal health over 6 months when used periodically for maintenance. Ongoing studies are looking at curcumin in peri-implantitis and aggressive periodontitis cases. Compared with chlorhexidine (CHX) adjuncts to scaling and root planing (SRP), local curcumin formulations typically show modest but statistically significant PPD/CAL improvements *versus* SRP alone, with outcomes broadly comparable to CHX in several trials (Wendorff-Tobolla et al., 2023a; Gupta et al., 2025; Zhang et al., 2021). Mechanistically, CHX acts *via* rapid bactericidal membrane disruption, whereas curcumin combines anti-biofilm/quorum-sensing interference, host-directed NF-κB/COX-2 attenuation, and, when used as a photosensitizer, reactive-oxygen generation (aPDT) (Lee et al., 2021). Curcumin is associated with fewer reports of tooth staining or taste disturbance than CHX, though high-quality comparative safety data are limited (Bossi et al., 2024); formulation heterogeneity and short follow-up warrant caution, and curcumin should be positioned as an investigational adjunct, not a replacement for CHX-based care (Zhang et al., 2021).

### 5.2 Oral mucositis trials

Over a dozen RCTs and several systematic reviews have examined curcumin for mucositis in cancer patients. A recent 2025 systematic review identified 12 trials (in patients undergoing radiotherapy/chemotherapy) and concluded that curcumin significantly reduces the risk, onset, and severity of oral mucositis across different cancer treatment regimens (Hao et al., 2025). For example, in head and neck cancer radiotherapy, meta-analyses show curcumin users are ∼50% less likely to develop severe mucositis (Grade 3–4) compared to controls (Zhang et al., 2021). In bone marrow transplant patients, curcumin mouthwash shortened mucositis duration by about 4–5 days on average *versus* placebo. These trials uniformly report improved oral pain scores and nutritional intake in curcumin groups (Bossi et al., 2024). The evidence here is strong enough that curcumin mouthwash is being incorporated into some standard care protocols for mucositis prevention. The trials also underscore curcumin’s safety: even in immunocompromised populations, curcumin did not predispose to infection or interfere with cancer therapy. Guideline-endorsed supportive options include benzydamine mouthwash and photobiomodulation alongside oral-care protocols (Elad et al., 2020). Curcumin (mouthwash/gel/nanocapsules) has reduced WHO grade and pain in randomized studies, but effect sizes vary with formulation and timing (Zanatta et al., 2010). Clinically, curcumin should be considered an adjunct within supportive-care pathways rather than a standalone therapy.

### 5.3 Recurrent aphthous stomatitis trials

Nine trials (469 patients) were summarized in a 2021 systematic review. The pooled data show curcumin treatment leads to faster healing of aphthous ulcers and greater pain reduction compared to placebo or no treatment, with results comparable to topical steroids in head-to-head comparisons (Al-Maweri et al., 2022). Approximately 70%–85% of patients receiving curcumin had significant improvement in pain within 3–4 days, *versus* 30%–40% of controls (Bakhshi et al., 2022). Ulcer size reduction was likewise more pronounced. There is moderate-quality evidence supporting curcumin as a first-line or adjunctive therapy in RAS, especially for patients with frequent or multiple ulcers. However, heterogeneity in formulations (rinses vs. gels) and treatment durations means future trials should standardize these parameters to strengthen recommendations (Deshmukh and Bagewadi, 2014).

### 5.4 Oral lichen planus trials

Clinical trials for OLP have yielded mixed results, as discussed. A 2024 meta-analysis of 10 studies found no overall significant benefit (Singh et al., 2023), while some individual RCTs did show that curcumin equals steroids in efficacy. The evidence quality is moderate to low, partly due to small sample sizes (most OLP trials have 20–40 patients per arm) and variations in outcome measures. Nonetheless, given curcumin’s safety and some positive signals, it is often considered a second-line option. Larger, multicenter trials are needed for OLP to reach a more definitive conclusion. At least one such trial is ongoing (testing nanocurcumin gel *versus* corticosteroid in erosive OLP with ∼100 patients). Standard care relies on topical corticosteroids (and calcineurin inhibitors in select cases). Curcumin has shown symptom relief and potential steroid-sparing effects in small trials; adequately powered, dose-standardized comparisons *versus* corticosteroids are needed before routine substitution (Amirchaghmaghi et al., 2016).

### 5.5 OSF and oral precancer trials

There have been at least 5 controlled trials in OSF and a few in leukoplakia. The aggregated evidence indicates curcumin (especially systemic plus topical) is efficacious in improving OSF symptoms and mouth opening, with several trials showing equivalence or superiority to standard treatments like intralesional steroids (Pan-On et al., 2022). One systematic review (Inchingolo et al., 2020) looking at natural antioxidants in OSF included curcumin trials and noted significant improvement in burning sensation and mouth opening in curcumin groups compared to baseline (Inchingolo et al., 2024). Curcumin also appeared to downstage the histopathological grading in some OSF cases (i.e., severe fibrosis turning to moderate). The level of evidence here is moderate; more large RCTs would bolster confidence. Given OSF’s prevalence in Southeast Asia, curcumin has been integrated into some routine management protocols there.

### 5.6 Anticaries/oral hygiene studies

While not as many RCTs exist for caries, the antiplaque efficacy of curcumin mouthwash has strong evidence (multiple trials vs. chlorhexidine). For caries prevention specifically, we rely on surrogate markers (plaque reduction, *S. mutans* counts). These surrogate outcomes all trend positively for curcumin (Mohajeri et al., 2021). One could extrapolate that sustained plaque control *via* curcumin would translate to lower caries incidence, but long-term caries trials are lacking. Nevertheless, at least one 1-year study in children saw fewer new caries with curcumin toothpaste use, suggesting a real-world benefit.

### 5.7 Oral cancer adjunct trials

Human data are early but promising. The Phase I trial of curcumin lozenges in oral cancer patients demonstrated biological activity (cytokine reduction) without toxicity, fulfilling its Phase I goals (Hegde et al., 2023). A follow-up Phase II (with curcumin + immunotherapy) will provide more insights into clinical efficacy. Additional evidence from microbiome modulation studies further suggests benefits (Gopalakrishna et al., 2023). Meanwhile, numerous *in vivo* studies and indirect clinical evidence (like curcumin’s effects in OSF, which is a cancer precursor) support that curcumin likely has beneficial anticancer effects. The evidence here is currently low-level in humans but high-level in mechanistic plausibility. Curcumin is investigational and not a replacement for surgery, radiotherapy, or chemotherapy. A randomized phase-1 window trial of a curcuminoid lozenge (APG-157) demonstrated salivary cytokine/microbiome modulation and systemic absorption, and the agent has received FDA Fast Track status; no survival or local-control benefit has been shown to date, so integration should occur only within clinical trials (Basak et al., 2020).

### 5.8 Safety profile across trials

It is noteworthy that across 80–100 studies on curcumin in oral conditions, no significant adverse events have been reported attributable to curcumin. Mild events like transient tongue yellowing (from the pigment) or a slight burning sensation (rarely) have been noted, but these are negligible compared to the side effects of many standard drugs. In high-dose systemic use (e.g., 6 g/day in the OLP study), some patients reported minor gastrointestinal upset, but even that was infrequent. This excellent safety, even in vulnerable patient groups, is a critical aspect of curcumin’s clinical appeal.

In evidence hierarchy, multiple meta-analyses and systematic reviews now exist–for periodontitis, mucositis, RAS, *etc.*, – most of which conclude that curcumin is effective and recommend its consideration in clinical practice. The strength of recommendation is strongest for conditions like gingivitis, periodontitis, and mucositis, where numerous high-quality RCTs concur on benefit. For others, like OLP and candidiasis, recommendations are cautious or suggest curcumin as an adjunct pending more data.

In summary, the accumulated clinical evidence positions curcumin as a versatile therapeutic adjunct in oral healthcare. It has been tested in a wide array of patient populations–from healthy individuals for plaque control to cancer patients for mucositis or tumor suppression–and shown beneficial outcomes with minimal risks. As research continues, we expect to see even more refined data (for example, dosage optimization, comparisons of curcumin formulations, and long-term effects). But even with current evidence, clinicians can feel reasonably confident in advising curcumin-based interventions as part of comprehensive oral disease management, aligning with patient preferences for natural remedies when appropriate.

## 6 Future directions and research perspectives

While extensive progress has been made in understanding and utilizing curcumin for oral health, several avenues remain for future exploration to fully realize its therapeutic potential. Below are key future directions and research considerations:.

### 6.1 Optimization of formulations and bioavailability

Despite numerous delivery innovations, enhancing the bioavailability and retention of curcumin in oral tissues continues to be a priority. Future research may focus on novel curcumin analogues that retain activity but have better solubility or stability (Bapat et al., 2023). For instance, analogues such as EF-24 or THC (tetrahydrocurcumin) could be tested in oral disease models. Additionally, controlled-release systems can be further refined, such as multilayer mucoadhesive patches that provide an initial burst of curcumin for pain relief, followed by sustained release for healing. Nanoformulations using dendrimers or stimuli-responsive nanoparticles (that release curcumin in response to pH or enzymes present at disease sites) are intriguing possibilities. The goal is to ensure adequate curcumin levels at the target site for a sufficient duration, thereby maximizing efficacy.

### 6.2 Large-scale clinical trials

Manycurrent clinical studies of the role of curcumin in oral health have relatively small sample sizes or single-center designs. To strengthen the evidence and potentially influence clinical guidelines, large, multicenter randomized trials are needed (Inchingolo et al., 2024; Gobbo et al., 2024). For example, a large trial in periodontal maintenance patients could compare curcumin gel vs. placebo over a year to determine differences in clinical attachment loss or tooth retention. In oral mucositis, a phase III trial of curcumin rinse vs. standard care across multiple cancer centers could pave the way for formal recommendations in oncology protocols (Morsy et al., 2023). Similarly, a sizable trial in recurrent aphthous stomatitis comparing curcumin, steroids, and placebo in a crossover design would be valuable to conclusively prove (or refute) the benefit of curcumin. Such trials should also standardize curcumin preparations (perhaps using pharmaceutical-grade bioavailable formulations) to make results generalizable.

### 6.3 Mechanistic studies in humans

While mechanistic insights abound from *in vitro* and animal studies, more translational research in humans would help clarify how curcumin works *in vivo* (Mukherjee and Krishnan, 2023). This could include tissue biopsies or saliva analyses from patients before and after curcumin treatment, and examination of changes in inflammatory mediators, gene expression profiles, and microbiome composition, *etc.* For example, analyzing gingival crevicular fluid in patients with periodontitis treated with curcumin might reveal reductions in specific cytokines or bone resorption markers (such as RANKL), solidifying the causal link between the molecular effects of curcumin and clinical improvements (Siyuan et al., 2023). In patients with OSCC taking curcumin, tumor tissue analysis could confirm the modulation of angiogenesis or immune cell infiltration as seen in preclinical models (Joshi et al., 2023). Biomarker studies such as these would bolster the mechanistic rationale for curcumin and could identify responders vs. nonresponders (perhaps some individuals have differences in curcumin metabolism or target expression that influence outcomes).

### 6.4 Synergistic therapies and combination approaches

Future research should explore how curcumin can be integrated with existing treatments for synergistic effects. For example, whether the combination of curcumin with a low-dose local antibiotic in periodontitis could yield additive benefits could be explored. Alternatively, in oral cancer, combining curcumin with immunotherapy or targeted therapy might improve response rates, as suggested by early trials. Curcumin might also synergize with other natural agents–a combined oral herbal rinse [e.g., curcumin with green tea (*Camellia sinensis* (L.) Kuntze; family Theaceae) extract or aloe vera (L.) Burm.f. (family Asphodelaceae)] might cover a broader spectrum of activity. Curcumin as a steroid-sparing agent is another area of research, such as the use of lower doses of steroids in OLP or OSF when curcumin is concurrently administered, to evaluate whether this reduces steroid side effects while maintaining efficacy. Combination regimens need careful design, but they could maximize patient outcomes by attacking diseases on multiple fronts. Table 4 outlines recent and ongoing studies exploring the use of curcumin in combination with other therapeutic modalities for oral diseases.

**TABLE 4:** Emerging strategies combining curcumin with other agents for synergistic effects in oral health applications. Combination therapies hold potential for enhancing efficacy while minimizing side effects.

Formulation type	Carrier material/system	Route/application	Target disease/use	Key advantages	References
Mouthwash/rinse	Hydroalcoholic solution, nanomicelles	Rinse 2–3× daily	Gingivitis, Mucositis	Easy access to the entire oral cavity, rapid onset, and nanomicelles bioavailability	Abdel-Fatah, Reham et al. BMC oral health vol. 2023, November 19; 23(1):883. (Abdel-Fatah et al., 2023)
Topical gel	Carbopol gel, Orabase, Chitosan gel	Direct lesion application	RAS, OLP, Post-SRP Healing	Sustained contact, lesion specificity, patient comfort	Idrees M. J Pers Med. J Pers Med. 2023 December 19; 14(1):1. (Idrees and Kujan, 2023)
Mucoadhesive film	Hydroxypropyl methylcellulose, Chitosan	Buccal mucosa, ulcer sites	OLP, Aphthous Ulcer	Prolonged mucosal adhesion, slow drug release, and minimal dosing frequency	Bapat RA et al. Environ Res. 2023 December 1; 238(Pt 1):116, 971. (Bapat et al., 2023)
Lozenges/troches	APG-157 lozenges (proprietary nanocurcumin)	Oral cavity absorption	Oral Cancer, OSF	Enhances salivary and tissue absorption, modulates tumor microenvironment	Inchingolo F, et al. Antioxidants (Basel). 2024 May28; 13(6):660. (Inchingolo et al., 2024)
Nanoparticles	Solid Lipid NP, Polymeric NP (PLGA), Nanoemulsions	Oral rinse or topical gel	Periodontitis, Biofilm infections	Cellular uptake, stability, and better plaque penetration	Bapat RA et al. Environ Res. 2023 December 1; 238(Pt1):116971. (Bapat et al., 2023)
Liposomal form	Phosphatidylcholine liposomes	Oral rinse	Oral Cancer, Mucositis	Lipid vesicle fusion with the mucosa improved tissue penetration	Dipalma G, et al. Antioxidants (Basel). 2024 September25; 13(10):1160. (Dipalma et al., 2024)
Chewing gum	Gum base with emulsified curcumin	Buccal absorption during chewing	Oral inflammation, Caries prevention	Constant mucosal release, patient adherence	Inchingolo F et al. Antioxidants (Basel). 2024 May28; 13(6):660. (Inchingolo et al., 2024)
Toothpaste	Herbal paste with curcumin	Twice daily brushing	Gingivitis, Caries	Daily exposure, antimicrobial and anti-inflammatory effects	Paradowska-Stolarz, Anna et al. Int J Mol Sci. 2021 September 25; 22(19):10,337. (Paradowska-Stolarz et al., 2021)

### 6.5 Personalized medicine and genomic considerations

Individuals may vary in their response to curcumin. Future studies might identify genetic polymorphisms that affect the metabolism of curcumin (such as in UDP-glucuronosyltransferases) or polymorphisms in inflammatory genes that make someone more or less responsive to the activity of curcumin (Kumar et al., 2022). This could pave the way for a personalized approach in which curcumin is recommended more strongly to patients who are predicted to benefit (e.g., those with high NF-κB activity profiles, or those with certain microbiome characteristics that curcumin can modulate). Tailoring treatment intensity, such as more frequent curcumin application for patients with severe oxidative stress markers, could also be explored.

### 6.6 Long-term use and chemoprevention

More data on the long-term use of curcumin in the oral cavity are needed. Can daily use of curcumin over the years prevent dental caries or periodontal progression? Does long-term use in patients with precancerous lesions reduce malignant transformation rates? Cohort or observational studies could address these questions (Viglianisi et al., 2024). For instance, tracking a group of patients with OSF or leukoplakia on long-term curcumin supplements *versus* a control group could yield insights into differences in cancer incidence over time. Similarly, a community-level study in which one group used a curcumin-infused oral care regimen and the other did not will show differences in the incidence of caries or gingivitis over a few years. These studies would help position curcumin not only as a treatment but also as a preventive or maintenance agent in oral health.

### 6.7 Addressing bioavailability concerns critically

Several experts have highlighted that curcumin may behave as a “Pan-Assay Interference Compound” (PAINS), raising concerns that some reported *in vitro* activities may reflect assay artefacts or nonspecific promiscuous binding rather than true target-specific effects (Bolz et al., 2021). This underscores the need to clearly separate *in vitro* findings from validated *in vivo* pharmacodynamics. Future research should critically evaluate whether sufficient active curcumin (or its metabolites) reaches oral or systemic target tissues at pharmacologically relevant concentrations in humans, and whether observed clinical benefits correlate with biologically plausible exposure–response relationships.

### 6.8 Regulatory approval pathways

As more evidence accumulates, efforts can be made to achieve regulatory endorsements for curcumin in specific indications (e.g., FDA or EMA approvals of a curcumin oral rinse as a medical device or drug for mucositis) (Batra et al., 2019). Future work may involve creating standardized, quality-controlled curcumin products and conducting the necessary trials for approval. This will ensure wider acceptance in mainstream practice.

### 6.9 Exploring new indications

There are oral conditions not yet well-studied with respect to the role of curcumin. For example, could curcumin mouthwash help in halitosis by reducing oral microbial load and volatile sulfur compound production? Alternatively, in Sjogren’s syndrome, where oral dryness and inflammation are issues, curcumin might aid minor salivary gland inflammation. Orthodontic treatment pain or ulceration is another area where a curcumin gel might soothe the mouth ulcers that sometimes occur with braces (Srivastava et al., 2018). These exploratory areas could open new uses for curcumin.

In conclusion, while the use of curcumin has already transitioned from the bench to the bedside in many respects, continuing research is vital to address the remaining questions and expand its utility. An emphasis on rigorous, large-scale trials, innovative delivery, and mechanistic clarity will help integrate curcumin more fully into evidence-based oral healthcare. In the future, a scenario in which curcumin (or improved derivatives) could become a routine component of dental and oral medicine practices, as a preventive supplement, a treatment adjunct, or even part of restorative materials is likely, contributing to a more holistic and natural approach to managing oral health and disease.

## 7 Conclusion

Curcumin has emerged as a multifaceted therapeutic agent in the realm of oral health, backed by a growing body of laboratory and clinical evidence. This comprehensive review has highlighted how the anti-inflammatory, antioxidant, and antimicrobial properties of curcumin converge to address the pathogenesis of numerous oral diseases (Wang et al., 2023). At the molecular level, curcumin modulates key pathways–from inhibiting NF-κB and proinflammatory cytokines to activating Nrf2-driven antioxidant defenses and disrupting microbial biofilms–thereby breaking the cycle of inflammation and oxidative stress that underlies conditions such as periodontitis, mucositis, and oral lesions (Grant et al., 2023; Girisa et al., 2021). Clinically, curcumin has demonstrated beneficial outcomes across a spectrum of applications: It reduces dental plaque and gingival inflammation comparable to conventional antiseptics, enhances the healing of periodontal pockets when used as an SRP adjunct, alleviates pain and accelerates ulcer resolution in recurrent aphthous stomatitis, provides symptomatic relief and possibly regression in oral precancerous conditions such as OSF, mitigates the severity of chemotherapy and radiotherapy-induced oral mucositis, and shows promise as an adjunct in oral cancer therapy by modulating the tumor microenvironment (Indriyani and Nur’aeny, 2025; Inchingolo et al., 2024).

A salient advantage of curcumin is its exceptional safety profile and patient acceptability. Over decades of study, it has consistently been shown to be nontoxic even at high doses and for prolonged use. Patients often prefer natural treatment options, and curcumin fulfills that desire without forcing a trade-off in efficacy; indeed, numerous trials have reported that it is as effective as standard drugs such as chlorhexidine mouthwash or corticosteroids in certain contexts (Tossetta et al., 2024). The integration of curcumin into various delivery systems–from gels and rinses to advanced nanoparticle carriers–has further enhanced its practicality and effectiveness in the oral cavity, ensuring that adequate concentrations reach the target tissues (Li et al., 2023).

Despite these successes, it is clear that curcumin is not a panacea, and it is most appropriately viewed as a valuable adjunct to conventional therapy rather than a wholesale replacement. Its relatively modest systemic bioavailability means that for systemic effects, formulation improvements or combination strategies are needed. Certain conditions (like advanced oral lichen planus or aggressive oral cancers) will still require conventional interventions, with curcumin serving to complement and improve outcomes rather than act alone. Furthermore, as highlighted in the Future Directions, continued research is necessary to optimize dosing, standardize formulations, and confirm long-term benefits in larger populations. Areas where evidence is mixed or limited remain, and these gaps should be filled by well-designed trials and mechanistic studies (Hashim et al., 2025). For clinical adoption, future studies should use standardized, GMP-compliant curcumin formulations with defined curcuminoid compositions and release/retention profiles, and report stability (ICH Q1) (Naicker et al., 2024), quality-by-design controls (ICH Q8/9/10) (Yu et al., 2014; Yang et al., 2025; VanDuyse et al., 2021), and batch specifications. We propose multicenter, adequately powered RCTs with harmonized endpoints [e.g., periodontitis: PPD/CAL (Loos and Needleman, 2020); mucositis: WHO/OMAS (Sonis et al., 1999; McGuire et al., 2013), embedded PK/retention substudies (Tmax, C]max, mucosal residence), and prospectively defined safety/pharmacovigilance plans (Ferreira-da-Silva et al., 2024). This framework will support regulatory review and, if effective, post-marketing surveillance.

Future research should prioritize standardized, regulator-ready curcumin formulations with defined curcuminoid compositions, release profiles, and mucosal retention (e.g., mucoadhesive films or nanomicelles), accompanied by stability and pharmacokinetic data as well as device–drug specifications for antimicrobial photodynamic therapy. Trials should preregister validated response biomarkers—such as salivary cytokines (IL-1β, IL-6, TNF-α), oxidative stress readouts (e.g., Nrf2 target expression, 8-OHdG), plaque/biofilm metrics, and digital image–based mucositis scores—and incorporate patient-reported outcomes and safety monitoring. Clinically, adequately powered, multicenter head-to-head RCTs should evaluate curcumin as an adjunct integrated into standard oral care regimens (e.g., SRP for periodontitis; guideline-concordant oral hygiene and supportive care for RT/CT-induced mucositis; perioperative pathways in OSCC), using standardized dosing, longer follow-up, and cost-effectiveness analyses to inform adoption.

Nonetheless, the current state of knowledge justifies the incorporation of curcumin into oral healthcare practices. For example, clinicians can recommend the use of curcumin mouthwash or gel for patients with gingivitis who seek herbal alternatives, or the use of curcumin gel as a postscaling medication in periodontal pockets to enhance healing. Oncologists and dentists working with patients with cancer can consider curcumin rinses to reduce the risk of oral mucositis and improve patient comfort. Oral medicine specialists might employ curcumin gel or lozenges in the management of aphthous ulcers, or OSF, or as a maintenance therapy in OLP patients is remission to possibly prolong disease-free intervals. The versatility of curcumin, spanning preventive to therapeutic roles, aligns well with the trend toward more holistic and patient-centered care in dentistry and medicine (Salehi et al., 2019). In conclusion, curcumin stands out as a natural “golden spice” transformed into a modern therapeutic goldmine for oral health. It exemplifies how a traditional remedy can be rigorously evaluated and adapted to contemporary clinical use. By targeting inflammation, oxidative stress, and microbes all at once, curcumin addresses the triad of factors common to many oral diseases. The evidence reviewed herein suggests that when used judiciously and in the right formulation, curcumin can significantly benefit patients by reducing disease burden, increasing quality of life, and potentially improving clinical outcomes in both routine and challenging oral health conditions. As research progresses, we anticipate even broader applications and more refined use of curcumin in oral medicine, solidifying its place in the integrative armamentarium for achieving and maintaining oral health.

Ultimately, curcumin’s journey in oral health–from ancient spice to scientifically validated adjuncts—emphasizes the importance of bridging traditional knowledge with rigorous science, and it highlights more natural, safe, and effective approaches for managing oral diseases in the future.

## Ethical statement

This study did not involve any experiments on humans or animals performed by any of the authors. For the sections involving clinical data, all procedures followed were in accordance with the ethical standards of the institutional and national research committees. Ethical approval was obtained from the Ethics Committee of Chongqing General Hospital and The Second Affiliated Hospital of Chongqing Medical University. No identifiable patient data were used in this manuscript.

## Glossary

ACSL4: Acyl-CoA Synthetase Long Chain Family Member 4
AI: Artificial Intelligence
AP-1: Activator Protein-1
aPDT: Antimicrobial Photodynamic Therapy
APG-157: Curcuminoid Botanical Lozenge (Investigational)
ARDS: Acute Respiratory Distress Syndrome
ARE: Antioxidant Response Element
AUC: Area Under the Receiver Operating Characteristic Curve
Bax: Bcl-2-Associated X Protein
Bcl-2: B-cell Lymphoma 2
BCM-95: Bio-Enhanced Curcumin Formulation
CAL: Clinical Attachment Level
CAT: Catalase
CD8+: Cluster of Differentiation 8-positive (cytotoxic T cells)
CHX: Chlorhexidine
COX-2: Cyclooxygenase-2
CT: Chemotherapy
CTGF: Connective Tissue Growth Factor
DAMPs: Damage-Associated Molecular Patterns
EGF: Epidermal Growth Factor
EHR: Electronic Health Record
EMT: Epithelial–Mesenchymal Transition
GI: Gingival Index
GPx: Glutathione Peroxidase
HIF-1α: Hypoxia-Inducible Factor 1-alpha
HO-1: Heme Oxygenase-1
HRV: Heart Rate Variability
ICU: Intensive Care Unit
IKK: IκB Kinase
IL-1β: Interleukin-1 beta
IL-6: Interleukin-6
IL-8: Interleukin-8
iNOS: Inducible Nitric Oxide Synthase
Keap1: Kelch-Like ECH-Associated Protein 1
LOS: Late-Onset Sepsis
LPS: Lipopolysaccharide
MAPK: Mitogen-Activated Protein Kinase
MIC: Minimum Inhibitory Concentration
ML: Machine Learning
MMP: Matrix Metalloproteinase
MyD88: Myeloid Differentiation Primary Response 88
NF-κB: Nuclear Factor kappa-light-chain-enhancer of activated B cells
NICU: Neonatal Intensive Care Unit
NP: Nanoparticle
NQO1: NAD(P)H Quinone Dehydrogenase 1
Nrf2: Nuclear Factor Erythroid 2–Related Factor 2
OLP: Oral Lichen Planus
OMAS: Oral Mucositis Assessment Scale
OMS: Oral Mucositis Scale (WHO)
OSCC: Oral Squamous Cell Carcinoma
OSF: Oral Submucous Fibrosis
PAMPs: Pathogen-Associated Molecular Patterns
PD-1: Programmed Cell Death Protein 1
PD-L1: Programmed Death-Ligand 1
PDT: Photodynamic Therapy
PI: Plaque Index
PLGA: Poly (lactic-co-glycolic acid)
PPAR-γ: Peroxisome Proliferator-Activated Receptor-gamma
PPD: Probing Pocket Depth
RANKL: Receptor Activator of Nuclear Factor Kappa-Β Ligand
RAS: Recurrent Aphthous Stomatitis
RCT: Randomized Controlled Trial
ROS: Reactive Oxygen Species
RT: Radiotherapy
S-AKI: Sepsis-Associated Acute Kidney Injury
SLC7A11: Solute Carrier Family 7 Member 11 (xCT)
SOD: Superoxide Dismutase
SRP: Scaling and Root Planing
STAT3: Signal Transducer and Activator of Transcription 3
T-regs: Regulatory T cells
TfR1: Transferrin Receptor 1
TGF-β: Transforming Growth Factor-beta
TLR: Toll-Like Receptor
TNF-α: Tumor Necrosis Factor alpha
VEGF: Vascular Endothelial Growth Factor
WHO: World Health Organization
